# *DOFT* and *DOFTIP1* affect reproductive development in the orchid *Dendrobium* Chao Praya Smile

**DOI:** 10.1093/jxb/erx400

**Published:** 2017-11-24

**Authors:** Yanwen Wang, Lu Liu, Shiyong Song, Yan Li, Lisha Shen, Hao Yu

**Affiliations:** Department of Biological Sciences and Temasek Life Sciences Laboratory, National University of Singapore, Singapore

**Keywords:** *Arabidopsis thaliana*, *DOFT*, *DOFTIP1*, flowering time, orchid, reproductive development

## Abstract

*FLOWERING LOCUS T* (*FT*) in Arabidopsis encodes the florigen that moves from leaves to the shoot apical meristem to induce flowering, and this is partly mediated by FT-INTERACTING PROTEIN 1 (FTIP1). Although FT orthologs have been identified in some flowering plants, their endogenous roles in Orchidaceae, which is one of the largest families of flowering plants, are still largely unknown. In this study, we show that *DOFT* and *DOFTIP1*, the orchid orthologs of *FT* and *FTIP1*, respectively, play important roles in promoting flowering in the orchid *Dendrobium* Chao Praya Smile. Expression of *DOFT* and *DOFTIP1* increases in whole plantlets during the transition from vegetative to reproductive development. Both transcripts are present in significant levels in reproductive organs, including inflorescence apices, stems, floral buds, and open flowers. Through successful generation of transgenic orchids, we have revealed that overexpression or down-regulation of *DOFT* accelerates or delays flowering, respectively, while alteration of *DOFT* expression also greatly affects pseudobulb formation and flower development. In common with their counterparts in Arabidopsis and rice, DOFTIP1 interacts with DOFT and affects flowering time in orchids. Our results suggest that while *DOFT* and *DOFTIP1* play evolutionarily conserved roles in promoting flowering, *DOFT* may have evolved with hitherto unknown functions pertaining to the regulation of storage organs and flower development in the Orchidaceae family.

## Teaser

*DOFT* and its interacting partner *DOFTIP1* promote flowering in the orchid *Dendrobium* Chao Praya Smile, and changes in the expression of *DOFT* affect pseudobulb formation and flower development.

## Introduction

The floral transition from vegetative to reproductive development is the most dramatic phase change in the life cycle of a flowering plant, and is tightly controlled by various flowering pathways in response to developmental and environmental signals. In the model plant *Arabidopsis thaliana*, convergence of the flowering signals from various pathways mediates the transcriptional regulation of several floral pathway integrators, including *FLOWERING LOCUS T* (*FT*) and *SUPPRESSOR OF OVEREXPRESSION OF CONSTANS 1* (*SOC1*), to precisely regulate the timing of the floral transition (Blázquez and Weigel, 2000; Lee *et al.*, 2000; Samach *et al.*, 2000; Li *et al.*, 2008). *FT* encodes a phosphatidylethanolamine binding protein and acts as a key flowering regulator that relays the photoperiod signal to activate floral meristem identity genes in Arabidopsis (Kardailsky *et al.*, 1999; Corbesier *et al.*, 2007). Overexpression of *FT* results in extremely early flowering, while *ft* mutants exhibit a late-flowering phenotype under long-day conditions (LDs) (Koornneef *et al.*, 1998). A major transcriptional regulator, CONSTANS (CO), in the photoperiod pathway activates *FT* mRNA expression in the vascular tissues of leaves under LDs (Samach *et al.*, 2000; An *et al.*, 2004; Wigge *et al.*, 2005).

Several studies have suggested that the FT protein acts as a mobile florigen signal moving from the leaves to the shoot apical meristem (SAM) to induce flowering (Corbesier *et al.*, 2007; Jaeger and Wigge, 2007; Mathieu *et al.*, 2007). In Arabidopsis, a multiple C2 domain and transmembrane-region protein (MCTP), FT-INTERACTING PROTEIN 1 (FTIP1), and a heavy-metal-associated (HMA) domain-containing protein, SODIUM POTASSIUM ROOT DEFECTIVE 1 (NaKR1), directly interact with FT and sequentially participate in the mediation of long-distance movement of FT from source leaves to the sink SAM in response to LDs (Liu *et al.*, 2012; Zhu *et al.*, 2016). FTIP1 is associated with FT in companion cells of the phloem and is specifically required for FT transport from companion cells to sieve elements through plasmodesmata (Liu *et al.*, 2012). NaKR1 is activated by CO under LDs, and regulates long-distance movement of FT from sieve elements in leaves to those below the SAM (Zhu *et al.*, 2016). In the SAM, it has been suggested that FT interacts with FD, a bZIP transcription factor, to directly activate the expression of *SOC1* and a floral meristem identity gene, *APETALA1* (*AP1*), thus initiating flower development (Abe *et al.*, 2005; Wigge *et al.*, 2005). Despite the progress made in understanding the flowering mechanisms involving FT and its interacting partners in Arabidopsis, the biological functions of their orthologs in orchids largely remain elusive.

Orchids belong to the family Orchidaceae, which is one of the largest families of angiosperms. As a group of important ornamental plants with great diversity and specialized floral morphology, this family is recognized as an economically important commodity in the international floriculture industry. They contribute to a large share of global floriculture trade as cut flowers and potted plants, partly because of their attractive flower morphology and long shelf life (Da Silva, 2013). In addition to their high economic value, orchids provide unique genetic material for the study of the mechanisms of plant reproductive development, such as flower development, pigment formation, and flower senescence, owing to their distinctive and colorful flower morphology as well as their specialized reproductive strategies (Da Silva *et al.*, 2014; Gutiérrez, 2010; Yu and Goh, 2001). A major obstacle for breeding and for the use of orchids in economic and academic applications is the prolonged vegetative phase, which makes it time-consuming to select desirable reproductive traits through traditional breeding methods (Da Silva *et al.*, 2014; Yu and Goh, 2001). Thus, it is important to understand the molecular mechanisms involved in the floral transition in orchids, so that the knowledge gained can be applied to classical breeding or to targeted manipulation of orchid varieties with desirable flowering traits.

In order to facilitate molecular studies of orchid development, we have previously developed a reproducible gene transformation coupled with an *in vitro* tissue culture system for the orchid *Dendrobium* Chao Praya Smile using L-methionine sulfoximine (MSO), as an agent for the selection of transgenic plants with the *bialaphos resistance* (*bar*) gene as a selectable marker (Yu and Goh, 2000*a*; Yu *et al.*, 2001; Chai *et al.*, 2007; Ding *et al.*, 2013). In this study, we isolated the orchid orthologs of *FT* and *FTIP1*, namely *DOFT* and *DOFTIP1*, respectively, from *Dendrobium* Chao Praya Smile, and characterized their biological functions in orchids utilizing the established gene transformation system. Expression analysis demonstrated that transcripts of both *DOFT* and *DOFTIP1* increased in orchid plantlets during the transition from vegetative to reproductive development. Expression of *DOFT* and *DOFTIP1* rescued the late-flowering phenotype of their corresponding Arabidopsis mutants, *ft-10* and *ftip1-1*, respectively. Through the creation of transgenic orchids, we further determined that alteration of *DOFT* expression in *Dendrobium* Chao Praya Smile not only significantly affected flowering time, but also influenced pseudobulb formation and flower development. In contrast, while DOFTIP1 interacted with DOFT, DOFTIP1 only affected flowering time in orchids. Therefore, our results suggest that although both *DOFT* and *DOFTIP1* are involved in promoting flowering, *DOFT* could have evolved to exert novel functions in regulating the development of pseudobulbs and floral organs in orchids.

## Materials and methods

### Plant material and growth conditions

*Dendrobium* Chao Praya Smile, a hybrid of *Dendrobium* Pinky and *Dendrobium* Kiyomi Beauty, were grown under long days (16 h light/8 h dark). Under our *in vitro* orchid culture system for *Dendrobium* Chao Praya Smile, calli that developed from seeds served as the starting material and were cultured at 24 °C under a 16-h photoperiod of 35 µmol m^–2^ s^–1^ from daylight fluorescent lamps as previously described (Ding *et al.*, 2013). *Arabidopsis thaliana* ecotype Columbia-0 (Col-0) plants were grown under long days at 23 ± 2 °C. The mutants used, *ft-10* and *ftip1-1*, are in the Col-0 background.

### Plant transformation

*Agrobacterium tumefaciens*-mediated transformation of Arabidopsis plants in the Col-0 background was carried out by a floral dipping method (Clough and Bent, 1998). All transgenic Arabidopsis plants generated were selected by Basta on soil. Genetic transformation of *Dendrobium* Chao Praya Smile was performed using particle bombardment or *A. tumefaciens*-mediated transformation coupled with the modified MSO selection system as previously reported (Chai *et al.*, 2007; Ding *et al.*, 2013).

### Cloning of *DOFT* and *DOFTIP1* from *Dendrobium* Chao Praya Smile

Total RNA was isolated from leaves of *Dendrobium* Chao Praya Smile using the RNeasy® Plant Mini Kit (Qiagen). Specific *FT*-like and *FTIP1*-like cDNA fragments were amplified with corresponding pairs of degenerate primers. The resulting cDNA fragments were cloned into the pGEM-T Easy vector (Promega) and sequenced. To further obtain the full-length sequences of the cDNAs, 3′-RACE and 5′-RACE were performed with gene-specific primers using the SMART^TM^ RACE cDNA Amplification Kit (BD Biosciences Clontech). The primers used for gene cloning are listed in Supplementary Table S1 at *JXB* online.

### Sequence analysis

Alignment of deduced amino acid sequences was carried out using the software MEGA 6.0 and BOXSHADE 3.21 (http://www.ch.embnet.org/software/BOX_form.html). The protein sequences of FT and FTIP1 orthologs aligned in this study were retrieved from the NCBI database (https://www.ncbi.nlm.nih.gov/). The phylogenetic tree was constructed with the Neighbor-joining algorithm using MEGA 6.0.

### Plasmid construction

To construct *35S:DOFT* and *35S:DOFTIP1*, the cDNAs of *DOFT* and *DOFTIP1* were amplified and ligated into pGreen 0229-35S containing 2 × 35S promoters. To create *DOFT* and *DOFTIP1* RNAi constructs, the amplified antisense- and sense-specific fragments for *DOFT* and *DOFTIP1* were ligated to the pGreen 0229 vector flanking a GUS fragment. To construct *AmiR-doft* and *AmiR-doftip1*, AmiRs were designed using the software on the WMD3 website (http://wmd3.weigelworld.org). Based on the *DOFT* and *DOFTIP1* sequences, sets of four primers for each gene were generated and used for the PCR amplification according to the published protocol (Schwab *et al.*, 2006). The resulting PCR fragments were cloned into the pGreen 0229-35S vector (Yu *et al.*, 2004).

### Southern blot analysis

Total DNA was isolated from leaves of *Dendrobium* orchids using the CTAB method (Porebski *et al.*, 1997). A 20-μg aliquot of genomic DNA was digested with different restriction enzymes, resolved on a 0.8% (w/v) agarose gel, and blotted onto a nylon membrane. The blot was hybridized overnight with the specific digoxigenin-labeled DNA, then washed and detected as previously described (Yu and Goh, 2000*b*).

### Expression analysis

Total RNA from either orchids or Arabidopsis was extracted using the FavorPrep Plant Total RNA mini-Kit (Favorgen) and reverse-transcribed using the M-MLV Reverse Transcriptase (Promega) according to the manufacturer’s instructions. Quantitative real-time PCR was performed on three biological replicates using the CFX384 Real-Time PCR Detection System (Bio-Rad) with the Maxima SYBR Green/ROX qPCR Master Mix (Fermentas). The orchid polyubiqutin gene *DOUbi* and the Arabidopsis *TUB2* gene were used as the normalization controls for expression analysis. Gene expression levels were calculated as previously described (Liu *et al.*, 2007). Primers used for quantitative real-time PCR are listed in Supplementary Table S1.

### *In situ* hybridization

Non-radioactive *in situ* hybridization was performed as previously described (Yu and Goh, 2000*b*). Gene-specific regions of *DOFT* and *DOFTIP1* were amplified and cloned into the pGEM-T Easy vector (Promega) to produce the templates for *in vitro* transcription using the DIG RNA Labeling Kit (Roche Molecular Biochemicals).

### Yeast two-hybrid assay

To construct the vectors for yeast two-hybrid assays, the coding sequence of *DOFT* was amplified and cloned into pGADT7, while the full-length and N-terminal regions of *DOFTIP1* were amplified and cloned into pGBKT7 (Clontech). The yeast two-hybrid assay was performed using the Yeastmaker Yeast Transformation System 2 (Clontech). The transformed cells were selected on SD–His/–Trp/–Leu medium supplemented with 3 mM 3-amino-1, 2, 4-triazole. β-galactosidase assays were performed according to the Yeast Protocols Handbook (Clontech).

### Glutathione S-transferase (GST) pull-down assay

The coding sequence of *DOFT* was cloned into the pGEX-4T-1 vector (Pharmacia) and transformed into *E. coli* Rosetta competent cells. Protein expression was induced by isopropyl-β-D-thiogalactoside at 16 °C. The soluble GST fusion proteins were extracted and immobilized on glutathione sepharose beads (Amersham Biosciences) for subsequent pull-down assays. The *DOFTIP1* N-terminal fragment containing the three C2 domains (N550) was cloned into the pGADT7 vector (Clontech). The resulting plasmid was added to the TNT T7 Quick Coupled Transcription/Translation System (Promega) to synthesize DOFTIP1 (N550)–HA (hemagglutinin) protein. The resulting fusion protein was then incubated with the immobilized GST and GST-FT fusion proteins. Proteins retained on the beads were resolved by SDS-PAGE and detected with anti-HA antibody (Santa Cruz Biotechnology).

### Bimolecular fluorescence complementation (BiFC) analysis

The full-length coding regions of *DOFT* and *DOFTIP1* were cloned into primary pSAT1 vectors. The resulting cassettes containing the fusion proteins driven by the constitutive promoters were cloned into a pGreen binary vector, pHY105, and transformed into *Agrobacterium*. These were in turn co-infiltrated into tobacco (*Nicotiana benthamiana*) leaves and observed through a confocal microscope.

### Co-immunoprecipitation experiment

Total proteins were isolated from transiently transformed tobacco leaves with a Plant Total Protein Extraction Kit (Sigma-Aldrich). The protein extracts were then immunoprecipitated using an anti-myc antibody (Santa Cruz Biotechnology). The total protein extracts as inputs and the immunoprecipitated proteins were resolved by SDS-PAGE and detected by an anti-HA antibody (Santa Cruz Biotechnology).

### Accession numbers

Sequence data from this article can be found in the GenBank/EMBL databases under the following accession numbers: *DOFT*, MF063061; *DOFTIP1*, MF063062; *DOAP1*, KY471451; *DOSOC1*, KC121576.

## Results

### Isolation of *DOFT* and *DOFTIP1* from *Dendrobium* Chao Praya Smile

To isolate putative *FT* and *FTIP1* orthologs from *Dendrobium* Chao Praya Smile, we designed degenerate primers from the conserved regions of *FT*-like and *FTIP1*-like genes, and amplified the cDNA fragments of around 400 bp and 750 bp, respectively. After confirming the sequence similarity between these fragments and the other orthologs of *DOFT* and *DOFTIP1*, we used the rapid amplification of cDNA ends (RACE) method to obtain the full-length cDNAs corresponding to these two fragments, designated *DOFT* and *DOFTIP1* (GenBank accession No. MF063061 and MF063062, respectively).

*DOFT* encodes a protein with 204 amino acid residues and an estimated molecular weight of 23 kDa. Multiple protein sequence alignment showed that *DOFT* exhibited 73, 79, and 94% identity to Arabidopsis FT, rice Hd3a, and another orchid FT ortholog OnFT from *Oncidium* Gower Ramsey, respectively (see Supplementary Fig. S1A). In particular, we found that Tyr85 and Gln140, which are the most critical residues for distinguishing FT and its closest homolog TERMINAL FLOWER1 (TFL1) in Arabidopsis (Hanzawa *et al.*, 2005; Ahn *et al.*, 2006), are present in the corresponding positions of Tyr111 and Gln166 of DOFT (Supplementary Fig. S1A). To determine the evolutionary relationship between DOFT and other FT orthologs, we constructed a phylogenetic tree that showed that DOFT was clustered in the FT clade and was closely related to OnFT as expected (Supplementary Fig. S1B). These results indicate that *DOFT* is the putative *FT* ortholog from *Dendrobium* Chao Praya Smile.

*DOFTIP1* encodes a protein with 815 amino acids and an estimated molecular weight of 92 kDa. Like other MCTPs in plants (Liu *et al.*, 2012, 2013), the deduced amino acid sequence of *DOFTIP1* contains three C2 domains and one PRT_C domain, which was predicted to be a membrane-targeted domain according to the topology analysis (see Supplementary Fig. S2A, B). Multiple sequence alignment revealed that DOFTIP1 shared 76% sequence identity with FTIP1, and even higher identity with FTIP1 orthologs in other monocots, such as OsFTIP1 in rice (83% identity; see Supplementary Fig. S2C) (Song *et al.*, 2017). Construction of a phylogenetic tree based on DOFTIP1 and other FTIP1-like proteins showed that DOFTIP1 was closely related to rice OsFTIP1 (Supplementary Fig. S2D). These sequence analyses also suggested that *DOFTIP1* is the putative ortholog of *FTIP1* in *Dendrobium* Chao Praya Smile.

To investigate the genomic organization of *DOFT* and *DOFTIP1* in *Dendrobium* orchids, we performed Southern blot analysis of genomic DNA digested with several enzymes using digoxigenin-labeled probes synthesized from the gene-specific regions of *DOFT* and *DOFTIP1*. DNA blot analysis revealed a single strong band for either *DOFT* or *DOFTIP1* (see Supplementary Fig. S2E), indicating that *DOFT* or *DOFTIP1* is present as a single-copy or low-copy number gene in the genome of *Dendrobium* Chao Praya Smile.

### Spatial and temporal expression patterns of *DOFT* and *DOFTIP1*

**Fig. 1.**
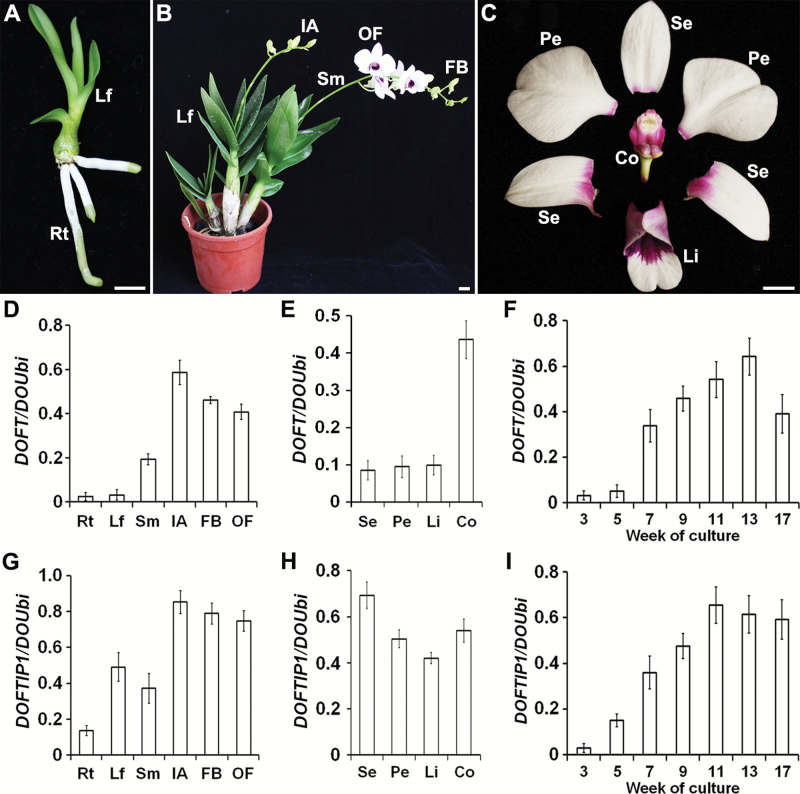
Quantitative analysis of *DOFT* and *DOFTIP1* expression in *Dendrobium* Chao Praya Smile. (A) A vegetative seedling. Rt, root; Lf, leaf. (B) A flowering plant with inflorescences bearing flowers at various stages. Lf, leaf; Sm, stem; IA, inflorescence apex; OF, open flower; FB, floral bud. (C) An open flower consisting of three sepals (Se), two petals (Pe), one lip (Li), and the reproductive organ column (Co). Scale bars in (A–C) =1 cm. (D–F) Quantitative analysis of *DOFT* expression in various tissues (D), different floral organs (E), and orchid plants at various developmental stages (F). Error bars indicate SD. (G–I) Quantitative analysis of *DOFTIP1* expression in various tissues (G), different floral organs (H), and orchid plants at different developmental stages (I). Error bars indicate SD. Expression levels in (D– I) were determined by quantitative real-time PCR analyses of three independently collected samples. The levels of gene expression were normalized to the expression of the orchid polyubiquitin gene (*DOUbi*).

To characterize the functions of *DOFT* and *DOFTIP1*, we first carried out quantitative real-time PCR to examine their spatial expression patterns in various orchid tissues harvested at normal greenhouse growth conditions (Fig. 1A–C and Supplementary Fig. S3A). The transcripts of both genes were detectable at low levels in vegetative tissues, such as roots, leaves, and stems, but were present at obviously higher levels in reproductive organs, such as inflorescence apices, floral buds, and open flowers (Fig. 1A, B, D, G). In the different floral organs (Fig. 1C), *DOFT* was highly expressed in the column (gynostemium, a fused structure of stigmas, styles, and stamens) (Fig. 1E), whereas *DOFTIP1* expression was high in all flower organs (Fig. 1H). In flowers at different development stages (see Supplementary Fig. S3A), expression levels of *DOFT* and *DOFTIP1* gradually decreased from young floral buds to open flowers (Supplementary Fig. S3B, C). These expression data imply that *DOFT* and *DOFTIP1* might be associated with reproductive organ development in *Dendrobium* Chao Praya Smile.

We further examined the temporal expression of *DOFT* and *DOFTIP1* in whole plants at various developmental stages under two growth conditions. In our established *in vitro* orchid culture system that allows rapid development of *Dendrobium* orchids from the vegetative to reproductive phase within about 3 months (Yu and Goh, 2000*a*; Ding *et al.*, 2013), thin sections of protocorms that serve as starting material generate protocorm-like bodies (PLBs) about 0.5 cm in length within 4 weeks, which further develop into vegetative plantlets in the subsequent 4 weeks. Most of these plantlets enter the floral transitional stage following another 4 weeks of culture, after which they produce visible inflorescences and flowers. We found that *DOFT* expression was low in 3-week-old PLBs and 5-week-old vegetative plantlets, but it dramatically increased in 7-week-old plantlets before the floral transitional stage (Fig. 1F). Its expression further increased in 9- and 11-week-old plantlets during the floral transitional stage, and reached a maximum level in 13-week-old plantlets that were undergoing reproductive development. *DOFTIP1* exhibited a temporal expression pattern similar to *DOFT*, but an obvious increase in its expression level was observed earlier in 5-week-old vegetative plantlets (Fig. 1I). In orchids grown under normal greenhouse conditions (see Supplementary Fig. S4A), the floral transition typically occurs 1 year after the starting material, which are young plantlets about 3 cm in height, are cultured in our greenhouse. We found that expression of both *DOFT* and *DOFTIP1* was low in 2- and 8-month-old vegetative plantlets, but dramatically increased in 15-month-old plantlets at the floral transitional stage, and remained high in plantlets at the subsequent reproductive stage (Supplementary Fig. S4B, C). These temporal patterns in the orchids grown under normal greenhouse conditions were consistent with those displayed in the orchids grown under the *in vitro* culture condition, suggesting that up-regulation of *DOFT* and *DOFTIP1* might be required for the floral transition in *Dendrobium*.

**Fig. 2.**
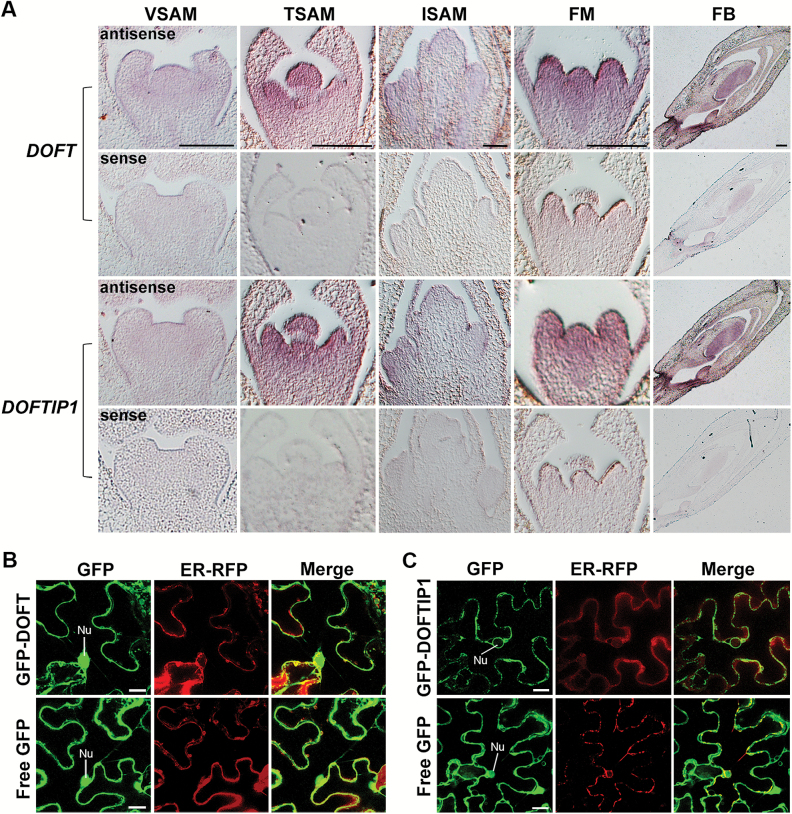
mRNA and protein localization of *DOFT* and *DOFTIP1.* (A) *In situ* localization of *DOFT* and *DOFTIP1* transcripts in *Dendrobium* Chao Praya Smile. Sections were hybridized with the gene-specific antisense and sense RNA probes of *DOFT* and *DOFTIP1*. VSAM, vegetative shoot apical meristem (6 weeks old); TSAM, transitional shoot apical meristem (12 weeks old); ISAM, inflorescence shoot apical meristem (16 weeks old); FM, floral meristem; FB, floral bud. Scale bars =200 μm. (B, C) Subcellular localization of GFP-DOFT (B) and GFP-DOFTIP1 (C) with free GFP in *N. benthamiana* leaf epidermal cells. Nu, nucleus. GFP, GFP fluorescence; ER-RFP, RFP fluorescence of an ER marker; Merge, merged images of GFP and RFP. Scale bars =20 μm.

As both *DOFT* and *DOFTIP1* were highly expressed in inflorescence apices and flowers (Fig. 1D, G), we then performed *in situ* hybridization using specific antisense *DOFT* and *DOFTIP1* RNA probes to investigate their detailed localization in shoot apices and flowers harvested under the *in vitro* culture system. *DOFT* and *DOFTIP1* expression was barely detectable in 6-week-old vegetative SAMs, but greatly increased in 12-week-old transitional SAMs (Fig. 2A). At the inflorescence and flower development stage, *DOFT* and *DOFTIP1* transcripts were distributed throughout the inflorescence SAM and the flanking floral primordia, floral meristems, and floral buds with developing floral organs (Fig. 2A). These observations, together with the quantitative expression data (Fig. 1), provide evidence that *DOFT* and *DOFTIP1* expression could be involved in the regulation of the floral transition and flower development in orchids.

To explore whether the expression of *DOFT* and *DOFTIP1* was influenced by photoperiod, their mRNA levels in leaves of 16-month-old orchids (grown under greenhouse conditions) at the floral transitional stage were analysed at 4-h intervals over a 24-h period under LDs and short-days (SDs). *DOFT* was expressed at stable levels under SDs, but exhibited a diurnal oscillation under LDs with a peak at Zeitgeber time (ZT)-8 (8 h after the beginning of the light period) (see Supplementary Fig. S5A). In contrast, *DOFTIP1* expression did not show an obvious circadian rhythm under either LDs or SDs (Supplementary Fig. S5B). These results suggest that, like their counterparts in Arabidopsis, day length affects the expression of *DOFT* but not *DOFTIP1* in orchids.

To assess the subcellular localization of DOFT and DOFTIP1, the coding sequences of *DOFT* and *DOFTIP1* were fused with the green fluorescent protein (GFP) driven by the cauliflower mosaic virus (CaMV) *35S* promoter. We transiently co-expressed *35S:GFP-DOFT* or *35S:GFP-DOFTIP1* with an endoplasmic reticulum (ER) marker (ER-RFP) in *N. benthamiana* leaf epidermal cells, and found that GFP-DOFT and GFP-DOFTIP were similarly co-localized with ER-RFP, whereas only the GFP-DOFT signal appeared in the nucleus (Fig. 2B).

### *DOFT* promotes flowering in Arabidopsis

To explore the biological role of *DOFT*, we created transgenic Arabidopsis plants in which *DOFT* was driven by the *35S* promoter. Among 29 independent *35S:DOFT* T1 transgenic plants created at the T1 generation, all lines flowered earlier than wild-type plants, with an average of 4.1 rosette leaves under LDs (see Supplementary Fig. S6A, B). We further overexpressed *DOFT* in *Arabidopsis ft-10* loss-of-function mutants to test whether it could complement the loss of *FT*. A total of 22 independent *ft-10 35S:DOFT* transgenic plants were obtained at the T1 generation, and all of them flowered even earlier than wild-type plants, demonstrating a complete rescue of the late-flowering phenotype of *ft-10* (Supplementary Fig. S6A, B). Semi-quantitative PCR analysis showed that *DOFT* expression was higher in both *35S:DOFT* and *35S:DOFT ft-10* transgenic lines with stronger early-flowering phenotypes than in those with weak phenotypes (Supplementary Fig. S6C), indicating that *DOFT* might have a dosage-dependent effect on promoting flowering in Arabidopsis.

Previous studies in Arabidopsis have demonstrated that *FT* is expressed in the phloem, and that its protein movement to the SAM through the phloem system contributes to floral induction (Takada and Goto, 2003; Corbesier *et al.*, 2007; Jaeger and Wigge, 2007; Mathieu *et al.*, 2007). To examine whether *DOFT* plays a similar role in the phloem, we expressed the *DOFT* coding sequence under the phloem-specific *SUCROSE TRANSPORTER 2* (*SUC2*) promoter in both wild-type and *ft-10* backgrounds. Like *35S:DOFT*, most of the *SUC2:DOFT* transgenic lines in both backgrounds displayed earlier flowering than the wild-type and *ft-10* plants (see Supplementary Fig. S7A, B), suggesting that *DOFT* acts in a manner similar to *FT* in the Arabidopsis phloem.

### *DOFT* promotes flowering in *Dendrobium* Chao Praya Smile

**Fig. 3.**
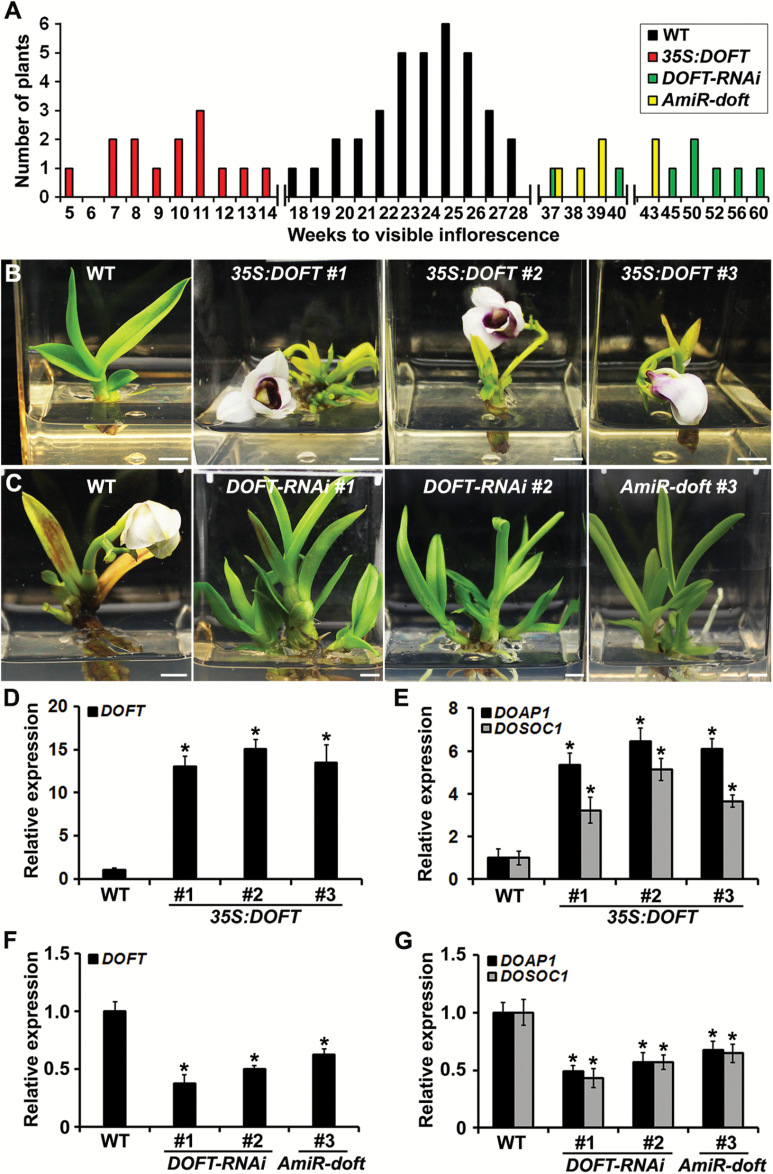
*DOFT* promotes flowering in *Dendrobium* Chao Praya Smile. (A) Comparison of flowering time of wild-type and various transgenic orchids. The orchid flowering time is represented by the number of weeks of culture until the first inflorescence stalk was visible. (B) Overexpression of *DOFT* (*35S:DOFT*) in *Dendrobium* Chao Praya Smile results in earlier flowering than wild-type plants at 10 weeks of *in vitro* culture. Scale bars = 1 cm. (C) Knock-down of *DOFT* by RNA interference (*DOFT-RNAi*) or artificial microRNA interference (*AmiR-doft*) in *Dendrobium* Chao Praya Smile causes later flowering than wild-type plants at 25 weeks of *in vitro* culture. Scale bars = 1 cm. (D, E) Quantitative analysis of the expression of *DOFT* (D), and *DOAP1* and *DOSOC1* (E) in wild-type and representative *35S:DOFT* transgenic orchids shown in (B) by real time RT-PCR. Error bars indicate SD. (F, G) Quantitative analysis of the expression of *DOFT* (F), and *DOAP1* and *DOSOC1* (G) in wild-type and representative *DOFT* knock-down transgenic orchids shown in (C) by real time RT-PCR. Error bars indicate SD. Total RNA extracted from leaves (D, F) or shoot apices (E, G) of orchid plants at 5 weeks of culture was used for expression analyses. The results in (D–G) were normalized against the expression levels of *DOUbi*, and the gene expression level in the wild-type plants was set as 1. Asterisks indicate significant differences in gene expression levels in various transgenic plants compared with those in corresponding wild-type plants (two-tailed paired Student**’**s *t*-test, *P*<0.05).

To understand the endogenous function of *DOFT* in *Dendrobium* orchids, we generated overexpression and knockdown transgenic orchids using particle bombardment or *Agrobacterium*-mediated transformation through an integrated gene transformation coupled with an *in vitro* tissue culture system (Yu and Goh, 2000*a*; Yu *et al.*, 2001; Chai *et al.*, 2007; Ding *et al.*, 2013). The presence of the transgenes in the putative transgenic orchids was examined by PCR genotyping and Southern blot analysis (see Supplementary Fig. S8). We created 14 independent *35S:DOFT* transgenic lines (Supplementary Fig. S8A, D), all of which generated the first visible inflorescence stalks at 5–14 weeks of culture (Fig. 3A, B). This was much earlier than the flowering time exhibited by wild-type orchids, which did not generate inflorescences until at least 18 weeks of culture (Fig. 3A). As expected, *DOFT* expression was much higher in leaves of the representative *35S:DOFT* lines than in the wild-type (Fig. 3B, D). In Arabidopsis, FT induces flowering through up-regulating other downstream flowering regulators, such as *SOC1* and *AP1*, in the shoot apex (Lee *et al.*, 2000; Abe *et al.*, 2005; Wigge *et al.*, 2005). Similarly, we found that the expressions of *DOSOC1* and *DOAP1* (Ding *et al.*, 2013; Sawettalake *et al.*, 2017), the orchid orthologs of *SOC1* and *AP1* in *Dendrobium* Chao Praya Smile, were significantly higher in shoot apices of *35S:DOFT* than in the wild-type (Fig. 3E).

We also created *DOFT* knockdown transgenic plants by RNA interference (RNAi) and artificial microRNA (AmiR) interference. Among 10 *DOFT*-*RNAi* and 6 *AmiR-doft* independent transgenic lines created (see Supplementary Fig. S8B–D), 14 lines generated the first visible inflorescence stalks at 37–60 weeks of culture (Fig. 3A, C), while the other two lines never generated any visible inflorescence stalks under our culture conditions. Thus, these *DOFT* knockdown transgenic plants displayed much later flowering time than wild-type orchids, which flowered at the latest at 28 weeks of culture (Fig. 3A). The expression of *DOFT*, *DOSOC1*, and *DOAP1* was consistently down-regulated in the representative knockdown lines compared to the wild-type (Fig. 3F, G). These observations suggest that *DOFT* plays a conserved role as *FT* in promoting orchid flowering, partially through engaging similar downstream regulators as revealed in Arabidopsis.

### *DOFT* affects other developmental processes in *Dendrobium* Chao Praya Smile

**Fig. 4.**
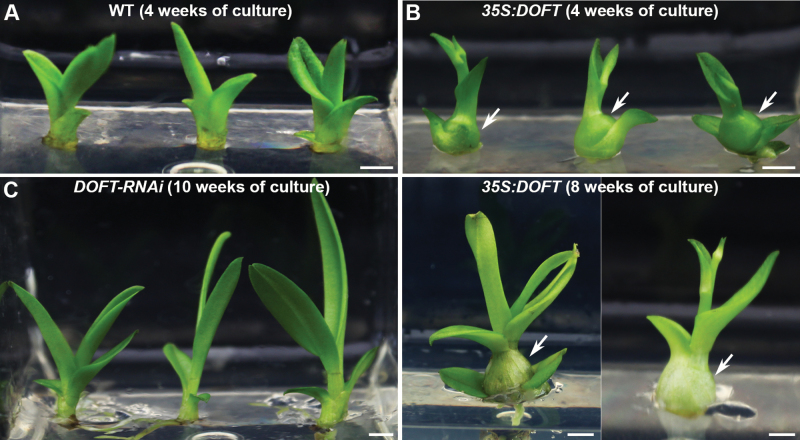
*DOFT* affects pseudobulb formation in *Dendrobium* Chao Praya Smile. (A) Pseudobulb formation does not occur in wild-type plants at 4 weeks of culture. (B) *35S:DOFT* transgenic orchid plants show obvious pseudobulb formation at 4 weeks (upper panel) and 8 weeks (lower panel) of culture. Arrows indicate pseudobulb formation. (C) Pseudobulb formation does not occur in *DOFT-RNAi* knock-down transgenic lines at 10 weeks of culture. Scale bars = 0.5 cm.

A marked morphological feature of *Dendrobium*, like many other epiphytic orchids, is the presence of a pseudobulb, which is a storage organ for supplying water, minerals, and carbohydrates (Zimmerman, 1990; Yong and Hew, 1995; Ng and Hew, 2000; He *et al.*, 2011). It has been suggested that pseudobulb photosynthesis recycles respiratory carbon, and that carbohydrate reserves in pseudobulbs contribute to the development of new shoots and inflorescences in the orchids *Catasetum viridiflavum* and *Oncidium* Goldiana (Zimmerman, 1990; Yong and Hew, 1995; Ng and Hew, 2000; Blanchard and Runkle, 2008; Wang *et al.*, 2008). Consistent with these previous findings, we found that pseudobulb formation usually occurred at 14 weeks under our *in vitro* tissue culture system for *Dendrobium* Chao Praya Smile at the beginning of the inflorescence and flower developmental stage (Yu and Goh, 2000*a*; Ding *et al.*, 2013), indicating that pseudobulb formation is required for reproductive development. In agreement with the early-flowering phenotype, half of the 14 *35S:DOFT* transgenic orchids produced visible pseudobulbs early at 4–6 weeks of culture, while the other half showed visible pseudobulbs at 8–12 weeks of culture (Fig. 4A, B). In contrast, pseudobulb formation in *DOFT*-*RNAi* and *AmiR-doft* transgenic lines was much delayed, either not occurring until 32 weeks of culture at the earliest or never occurring at all (Fig. 4C). These observations suggest that *DOFT* promotes both pseudobulb formation and flowering in *Dendrobium* Chao Praya Smile.

**Fig. 5.**
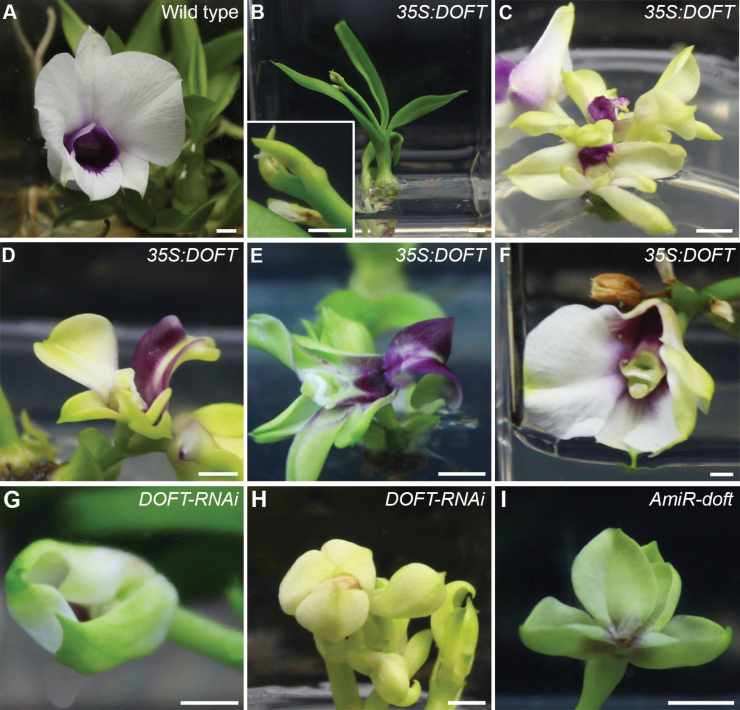
Alteration of *DOFT* expression compromises normal inflorescence and flower development in *Dendrobium* Chao Praya Smile. (A) A wild type orchid line. (B) A *35S:DOFT* transgenic orchid line generates an aborted inflorescence apex, shown enlarged in the inset. (C–I) Various *DOFT* transgenic orchid lines, including *35S:DOFT* (C–F), *DOFT-RNAi* (G, H), and *AmiR-doft* (I), generate flowers with either incomplete or abnormal floral organs. Scale bars: (A–F) = 0.5 cm, (G–I) = 1 cm.

We further investigated the effects of *DOFT* on flower phenotypes under the *in vitro* tissue culture system, and found that changes in *DOFT* expression in both overexpression (*35S:DOFT*) and knockdown (*DOFT*-*RNAi* and *AmiR-doft*) transgenic orchids caused a higher percentage of abnormal or incomplete inflorescences and floral organs than in the wild-type (Fig. 5 and Supplementary Table S2). These results, together with high expression of *DOFT* in reproductive organs, including inflorescence apices, floral buds, and open flowers (Fig. 1D), strongly suggest that in addition to control of flowering time, *DOFT* is also required for inflorescence and floral development in *Dendrobium* orchids.

### *DOFTIP1* accelerates flowering in Arabidopsis and *Dendrobium* Chao Praya Smile

In Arabidopsis, FTIP1 interacts with FT in companion cells and mediates its transport from companion cells to sieve elements through plasmodesmata (Liu *et al.*, 2012). Notably, either loss or overexpression of *FTIP1* causes a late-flowering phenotype, as the former compromises FT transport from the companion cells to sieve elements, whereas the latter deregulates FT protein transport out of the phloem system. To understand the biological function of *DOFTIP1*, we also created *35S:DOFTIP1* transgenic Arabidopsis plants in both wild-type and *ftip1-1* backgrounds. Similar to *35S:FTIP1* (Liu *et al.*, 2012), 17 out of 25 *35S:DOFTIP1* transgenic lines flowered later than wild-type plants under LDs (see Supplementary Fig. S9A–C). In contrast, overexpression of *FTIP1* in *ftip1-1* evidently rescued the late-flowering phenotype of *ftip1-1*, and 13 out 14 *ftip1-1 35S:DOFTIP1* lines displayed a comparable flowering time to the wild-type plants (Supplementary Fig. S9A–C). Thus, *DOFTIP1* is able to play a similar role to *FTIP1* in promoting flowering in Arabidopsis, but its expression in excessive amounts in wild-type plants could similarly deregulate FT transport, thus resulting in late flowering.

**Fig. 6.**
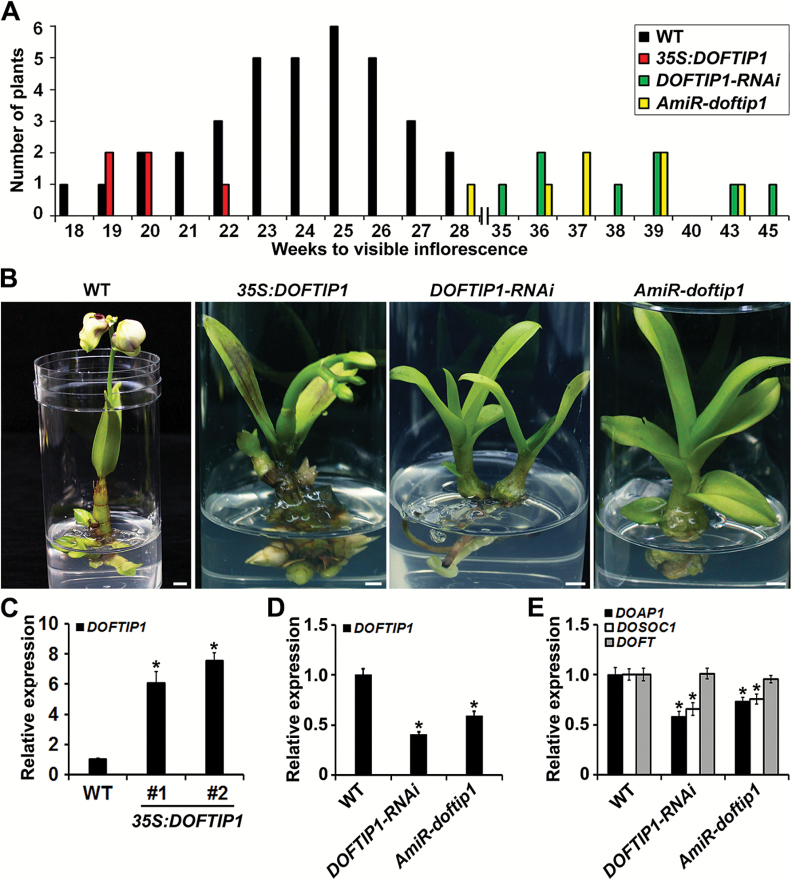
Down-regulation of *DOFTIP1* delays flowering in *Dendrobium* Chao Praya Smile. (A) Comparison of flowering time of wild-type and various transgenic orchids. (B) Knock-down of *DOFTIP1* by RNA interference (*DOFTIP1-RNAi*) or artificial microRNA interference (*AmiR-doftip1*) causes later flowering than wild-type and *35S:DOFTIP1* orchid plants at 25 weeks of *in vitro* culture. Scale bars = 1 cm. (C, D) Quantitative analysis of *DOFTIP1* expression in leaves of representative *35S:DOFTIP1* (C) and *DOFTIP1* knock-down (D) transgenic orchids by real time RT-PCR. Error bars indicate SD. (E) Quantitative analysis of the expression of *DOFT*, *DOAP1*, and *DOSOC1* in *35S:DOFTIP1* and *DOFTIP1* knock-down transgenic orchids by real time RT-PCR. Total RNA extracted from shoot apices of orchid plants at 10 weeks of culture was used for expression analysis. Error bars indicate SD. The results in (C–E) were normalized against the expression levels of *DOUbi*, and the expression level of each gene in the wild-type plants was set as 1. Asterisks indicate significant differences in gene expression levels in various transgenic plants compared with those in corresponding wild-type plants (two-tailed paired Student**’**s *t*-test, *P*<0.05).

We further created *DOFTIP1* overexpression and knockdown transgenic orchids to investigate the endogenous function of *DOFTIP1*. After PCR genotyping and Southern blot analysis (Supplementary Fig. S10), we obtained five, eight, and seven independent lines for *35S:DOFTIP1*, *DOFTIP1-RNAi*, and *AmiR-doftip1*, respectively. All the *35S:DOFTIP1* lines exhibited a comparable flowering time to the wild-type orchids, although *DOFTIP1* was overexpressed in the leaves of these transgenic lines (Fig. 6A–C). In contrast, most of the *DOFTIP1-RNAi* and *AmiR-doftip1* lines generated the first visible inflorescence stalks at 35–45 weeks of culture, which was much later than the flowering time of the wild-type (Fig. 6A, B). *DOFTIP1* expression was consistently down-regulated in leaves of the representative *DOFTIP1-RNAi* and *AmiR-doftip1* lines (Fig. 6D). Furthermore, we found that while down-regulation of *DOFTIP1* did not affect *DOFT* transcript levels, *DOSOC1* and *DOAP1* were significantly decreased in shoot apices of the *DOFTIP1-RNAi* and *AmiR-doftip1* lines compared to their expression in wild-type orchids (Fig. 6E). These results suggest that *DOFTIP1* promotes orchid flowering, but does not directly affect *DOFT* expression levels.

### *DOFTIP1* interacts with *DOFT*

**Fig. 7.**
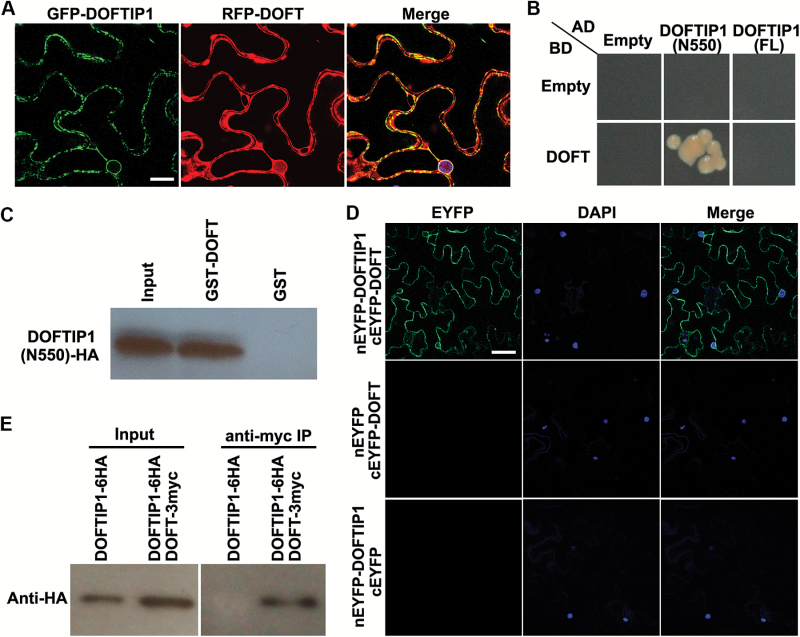
DOFTIP1 interacts with DOFT. (A) Co-localization of GFP-DOFTIP1 and RFP-DOFT in *N. benthamiana* leaf epidermal cells. Merge indicates merged images of GFP and RFP. Scale bar = 20 μm. (B) Yeast two-hybrid assay of the interaction between DOFT and the full-length DOFTIP1 (FL) and its N-terminus (amino acids 1–550; N550). Transformed yeast cells were grown on SD–His/–Trp/–Leu medium supplemented with 3 mM 3-amino-1,2,4-triazole. Empty refers to AD- or BD-containing vector only. (C) *In vitro* pull-down assay of the interaction between DOFT and DOFTIP1 (N550). ‘Input’ indicates 5% of HA-labeled DOFTIP1 (N550) subjected to pull-down by GST and GST-DOFT. (D) BiFC analysis of the interaction between DOFTIP1 and DOFT. DAPI, fluorescence of 4’,6-diamino-2-phenylindol; EYFP, fluorescence of enhanced yellow fluorescent protein; Merge, merged images of DAPI and EYFP. Scale bar = 10 μm. (E) *In vivo* interaction between DOFTIP1 and DOFT shown by co-immunoprecipitation. *35S:DOFTIP1-6HA* and *35S:DOFT-3myc* were transiently transformed into tobacco leaves, and total protein extracts were immunoprecipitated using an anti-myc antibody. The input and co-immunoprecipitated protein were detected using an anti-HA antibody.

Our findings on the similar tissue expression patterns of *DOFT* and *DOFTIP1* and their same roles in promoting flowering in both Arabidopsis and orchids prompted us to investigate whether they interact with each other, like their counterparts in Arabidopsis (Liu *et al.*, 2012). To this end, we examined the protein interaction between DOFTIP1 and DOFT through a few approaches. First, we co-expressed *35S:RFP-DOFT* and *35S:GFP-DOFTIP1* in *N. benthamiana* leaf epidermal cells, and found both RFP-DOFT and GFP-DOFTIP1 were co-localized to ER connected to the nuclear envelope (Figs 2B, C and 7A). Second, yeast two-hybrid assays further revealed that the truncated DOFTIP1 protein devoid of the PRT_C domain, DOFTIP1 (N550), interacted with DOFT, whereas no interaction was detected between the full-length DOFTIP1 and DOFT (Fig. 7B). Quantification of the yeast two-hybrid interaction by β-galactosidase assays confirmed the strong interaction between DOFTIP1 (N550) and DOFT (see Supplementary Fig. S11A). As the PRT_C domain could be a membrane-targeted domain (Supplementary Fig. S2A, B), the full-length DOFTIP1 protein might not be in the membrane-bound state in yeast cells. This may cause an appropriate folding of DOFTIP1 to prevent its interaction with DOFT. Third, a GST pull-down assay also demonstrated that HA-labeled DOFTIP1 (N550) bound to GST-DOFT, but not GST, induced in *E. coli* cells (Fig. 7C and Supplementary Fig. S11B). To test the *in planta* interaction between DOFTIP1 and DOFT, we further performed BiFC assays, and also found an enhanced YFP (EYFP) signal in *N. benthamiana* leaf epidermal cells except the nuclei (Fig. 7D). To verify this *in planta* interaction, we transiently transformed *35S:DOFTIP1-6HA* and *35S:DOFT-3myc* into tobacco leaves. Co-immunoprecipitation analysis of the protein extracts revealed that DOFTIP1-6HA interacted with DOFT-3myc only in the tobacco leaves transformed with both *35S:DOFTIP1-6HA* and *35S:DOFT-3myc* (Fig. 7E), substantiating the interaction between DOFTIP1 and DOFT in plant cells.

## Discussion

The proteins encoded by *FT* in Arabidopsis and its orthologs in several plant species have been identified as part of the mobile florigenic signals and play an important role in promoting flowering (Corbesier *et al.*, 2007; Jaeger and Wigge, 2007; Lin *et al.*, 2007; Mathieu *et al.*, 2007; Tamaki *et al.*, 2007). Although *FT*-like genes have also been isolated in a few orchid varieties (Hou and Yang, 2009; Huang *et al.*, 2012; Li *et al.*, 2012), these studies were unable to address their endogenous functions because of a lack of transgenic material. Thus, the biological functions of *FT* orthologs in the Orchidaceae, which is one of the largest families of flowering plants, are to date still largely unknown. In this study, we have characterized the functions of the orchid orthologs of *FT* and *FTIP1*, namely *DOFT* and *DOFTIP1*, respectively, from *Dendrobium* Chao Praya Smile, using an established gene transformation coupled with an *in vitro* tissue culture system. Our findings suggest that while both *DOFT* and *DOFTIP1* play conserved roles in promoting flowering, *DOFT* has evolved with hitherto unknown functions in regulating the development of pseudobulbs and floral organs in orchids.

Our results provide several pieces of evidence suggesting that *DOFT* is the *FT* ortholog in *Dendrobium* Chao Praya Smile. First, DOFT shares high sequence similarity with FT and it orthologs in other plant species, including the signature residues Tyr85 and Gln140 of FT in the corresponding positions (see Supplementary Fig. S1A). Phylogenetic analysis based on the full-length amino acid sequences also demonstrated that DOFT is clustered with another orchid *FT* ortholog, *OnFT*, from *Oncidium* Gower Ramsey, in the FT clade (Supplementary Fig. 1B). Second, *DOFT* expression gradually increased during the floral transition in *Dendrobium* orchids under both *in vitro* culture and normal greenhouse conditions (Fig. 1F and Supplementary Fig. S4). This is similar to the *FT* expression pattern in Arabidopsis (Kardailsky *et al.*, 1999; Kobayashi *et al.*, 1999). Third, overexpression of *DOFT* caused early flowering and completely rescued the late-flowering phenotype of *ft-10* in Arabidopsis (Supplementary Fig. S6). In addition, expression of *DOFT* driven by the phloem-specific *SUC2* promoter in Arabidopsis exhibited a promotive effect on flowering similar to overexpression of *DOFT* (Supplementary Fig. S7). These results suggest that *DOFT* plays the same role as *FT* in promoting flowering in the Arabidopsis phloem. Lastly, overexpression or down-regulation of *DOFT* accelerated or delayed flowering in *Dendrobium* Chao Praya Smile (Fig. 3), demonstrating that *DOFT* is indeed required for orchid flowering. Notably, *DOFT* expression was positively correlated with the expression of *DOSOC1* and *DOAP1*, the orthologs of *SOC1* and *AP1*, respectively, in *Dendrobium* Chao Praya Smile (Ding *et al.*, 2013; Sawettalake *et al.*, 2017). This is consistent with the effect of FT in up-regulating *SOC1* and *AP1* in Arabidopsis (Lee *et al.*, 2000; Abe *et al.*, 2005; Wigge *et al.*, 2005), implying that DOFT and FT may similarly control comparable downstream genes to promote flowering in *Dendrobium* Chao Praya Smile and Arabidopsis, respectively.

In Arabidopsis and rice, FTIP1 and its rice counterpart OsFTIP1 mediate transport of FT and its rice ortholog, RICE FLOWERING LOCUS T 1 (RFT1), respectively, from companion cells to sieve elements in the phloem, thus affecting their movement from leaves to the shoot apex (Liu *et al.*, 2012; Song *et al.*, 2017). In *Dendrobium* Chao Praya Smile, the orchid ortholog of FTIP1, DOFTIP1, exhibited several characteristics similar to FTIP1. In addition to sequence similarity between DOFTIP1 and FTIP1 or OsFTIP1, expression of *DOFTIP1* rescued the late-flowering phenotype of its corresponding Arabidopsis mutant *ftip1-1* (see Supplementary Fig. S9), while down-regulation of *DOFTIP1* delayed flowering in *Dendrobium* orchids (Fig. 6), indicating that *DOFTIP1* plays a similar role to *FTIP1* in promoting flowering. Both RFP-DOFT and GFP-DOFTIP1 were co-localized to the ER in plant cells (Fig. 7A). Furthermore, *DOSOC1* and *DOAP1* were similarly down-regulated in shoot apices of both *DOFT* and *DOFTIP1* knockdown orchids, whereas down-regulation of *DOFTIP1* did not affect *DOFT* expression (Figs 3G and 6E), implying that *DOFTIP1* may interact with *DOFT* at the protein level, thus affecting their common downstream genes. Indeed, several approaches confirmed the interaction between DOFTIP1 and DOFT *in vitro* and *in planta* (Fig. 7B–D). These observations all support the view that DOFTIP1 is the orchid counterpart of FTIP1 and interacts with DOFT to affect flowering time in *Dendrobium* Chao Praya Smile.

While DOFT plays an evolutionarily conserved role in promoting flowering in orchids, our findings have also revealed two interesting and hitherto unknown functions of *FT*-like genes in orchids. Firstly, *DOFT* affected the generation of pseudobulbs, which are a type of storage organ that contribute to reproductive development in many epiphytic orchids (Zimmerman, 1990; Yong and Hew, 1995; Ng and Hew, 2000; He *et al.*, 2011). High expression of *DOFT* promoted pseudobulb formation, which was, however, delayed when *DOFT* was down-regulated (Fig. 4). As pseudobulb formation evidently appears after the floral transition in *Dendrobium* Chao Praya Smile (Yu and Goh, 2000*a*; Ding *et al.*, 2013), it is highly possible that DOFT promotes flowering partly through positively regulating the formation of pseudobulbs so that these storage organs can provide sufficient quantities of the water, minerals, and carbohydrates that are essential for inflorescence and flower development in orchids. The effects of *DOFT* on pseudobulb formation and flowering in orchids is reminiscent of the similar role of *FT*-like genes in controlling flowering and tuberization in potato (Navarro *et al.*, 2011), indicating that *FT*-like genes may play broader roles in regulating other developmental processes required for the floral transition.

Secondly, *DOFT* also contributes to inflorescence and flower development in *Dendrobium* Chao Praya Smile. Alteration in *DOFT* expression levels in *35S:DOFT*, *DOFT*-*RNAi*, and *AmiR-doft* transgenic orchids resulted in more abnormal or incomplete inflorescences and floral organs than in wild-type plants (Fig. 5 and Supplementary Table S2), suggesting that an appropriate control of *DOFT* expression in inflorescences and developing floral organs is important for normal reproductive development in orchids. We consistently found that *DOFT* was expressed at high levels in reproductive organs, such as inflorescence apices, floral buds, and open flowers (Fig. 1D). Notably, among all the floral organs, *DOFT* was highly expressed in the column, which is a fused structure of reproductive organs, including stigmas, styles, and stamens (Fig. 1E). In agreement with this result, incomplete flowers without the column were often observed in *DOFT* knockdown lines, such as *AmiR-doft* (Fig. 5I), indicating that *DOFT* expression in the column is essential for reproductive organ development in orchids. In contrast to *DOFT*, changes in *DOFTIP1* expression did not greatly affect inflorescence and flower development compared to the wild-type, suggesting that *DOFTIP1* has a specific role in regulating flowering time rather than inflorescence and flower development in orchids.

To date, a number of *FT* orthologs have been isolated from a wide range of plant species. Besides the functional diversity of *DOFT*, as discussed above, some other *FT* orthologs have also been shown to have unique functions in various plant developmental processes; for example, *PtFT1* and *PtFT2* participate in SD-induced growth cessation and bud set in *Populus* (Böhlenius *et al.*, 2006; Hsu *et al.*, 2006), while *SFT* regulates fruit set, termination of sympodial meristems, and leaf architecture in tomato (Lifschitz *et al.*, 2006). These observations demonstrate that *FT*-like genes not only encode important mobile flowering signals, but also participate in other diverse developmental processes that need to be further elucidated.

## Supplementary data

Supplementary data are available at *JXB* online.

Fig. S1. Sequence analysis of *DOFT*.

Fig. S2. Sequence analysis of *DOFTIP1*.

Fig. S3. Expression of *DOFT* and *DOFTIP1* in flowers at different developmental stages.

Fig. S4. Quantitative analysis of temporal expression of *DOFT* and *DOFTIP1* in *Dendrobium* Chao Praya Smile grown under normal greenhouse conditions.

Fig. S5. Quantitative analysis of *DOFT* and *DOFTIP1* expression levels in leaves of *Dendrobium* Chao Praya Smile within a 24-h cycle under long and short days.

Fig. S6. Overexpression of *DOFT* promotes flowering in Arabidopsis.

Fig. S7. Expression of *SUC2:DOFT* promotes flowering in Arabidopsis.

Fig. S8. Molecular identification of *DOFT* transgenic orchids.

Fig. S9. Overexpression of *DOFTIP1* rescues the late flowering phenotype of Arabidopsis *ftip1-1*.

Fig. S10. Molecular identification of *DOFTIP1* transgenic orchids.

Fig. S11. Evidence that DOFTIP1 interacts with DOFT.

Table S1. List of primers used in this study.

Table S2. Comparison of flower development in wild-type and transgenic *Dendrobium* Chao Praya Smile after *in vitro* culture under our growth conditions.

## Supplementary Material

supplementary_Figures_Tables
