# Supplementary material for: DOFT and DOFTIP1 affect reproductive development in the orchid Dendrobium Chao Praya Smile
Source: J Exp Bot. 2017 Nov 24;68(21-22):5759–72. doi: 10.1093/jxb/erx400 (PMC5854133; doi:10.1093/jxb/erx400)
Supplement: supplementary_Figures_Tables [file erx400_suppl_supplementary_figures_tables.pdf]

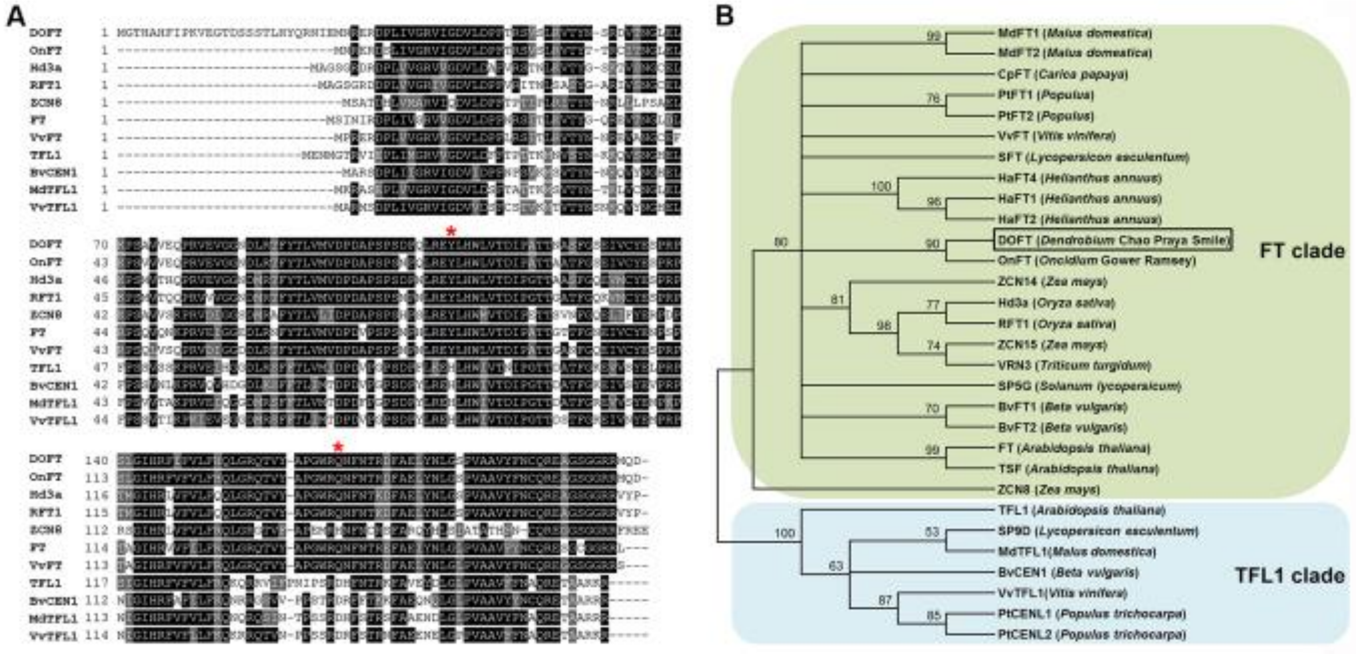

Supplementary Figure S1. Sequence analysis of *DOFT*. (A) Alignment of amino acid sequences of DOFT and other TFL1/FT family proteins. Sequences of OnFT (*Oncidium* Gower Ramsey), ZCN8 (*Zea mays*), Hd3a (*Oryza sativa*), RFT1 (*Oryza sativa*), VvFT (*Vitis vinifera*), VvTFL1 (*Vitis vinifera*), MdTFL1 (*Malus x domestica*), BvCEN1 (*Beta vulgaris*), TFL1 (*Arabidopsis thaliana*), and FT (*Arabidopsis thaliana*) were obtained from NCBI. Black and grey boxes indicate identical and similar residues, respectively. Red asterisks indicate Tyr111 and Gln166 of DOFT, which correspond to the critical residues that distinguish FT and TFL1 in Arabidopsis. (B) Phylogenetic analysis of DOFT and other TFL1/FT family proteins. The phylogenetic tree was generated with MEGA6 using the Neighbor-joining algorithm. Numbers on the major branches indicate bootstrap values (>50%) in 10,000 replicates.

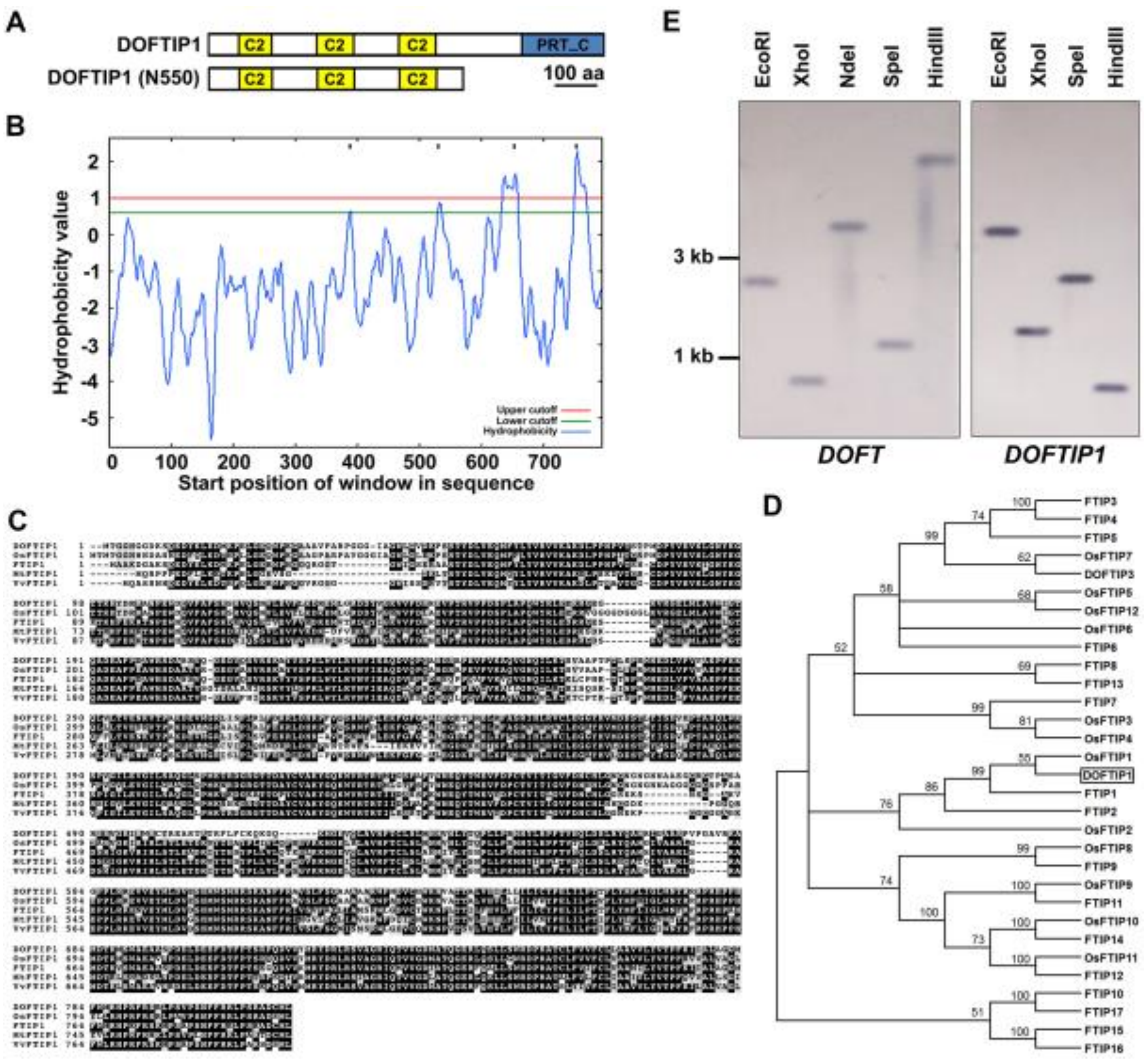

Supplementary Figure S2. Sequence analysis of *DOFTIP1*. (A) Schematic diagram shows three C2 domains and one PRT_C domain in the DOFTIP1 protein. The truncated form of DOFTIP1 (N550) devoid of the PRT_C domain was used for protein interaction studies shown in Fig. 6B, C. (B) Topology prediction of the transmembrane region in DOFTIP1 using the TopPred program. (C) Alignment of amino acid sequences of DOFTIP1 and other FTIP1-like proteins. Sequences of DOFTIP1 (*Dendrobium* Chao Praya Smile), OsFTIP1 (*Oryza sativa*), FTIP1 (*Arabidopsis thaliana*), MtFTIP1 (*Medicago truncatula*), and VvFTIP1 (*Vitis vinifera*) were obtained from NCBI. Sequence alignment was generated using ClustalW2 (EMBL-EBI). Black and grey boxes indicate identical and similar residues, respectively.(D) Phylogenetic analysis of DOFTIP1 and its orthologs in Arabidopsis (FTIP1-17) and rice (OsFTIP1-12). The phylogenetic tree was generated with MEGA6 using the Neighbor-joining algorithm. Numbers on the major branches indicate bootstrap values (>50%) in 10,000 replicates. (E) Southern blot analysis of *DOFT* and *DOFTIP1* genomic organization in *Dendrobium* Chao Praya Smile. DNA gel blots containing 20 µg of genomic DNA digested with enzymes indicated above the blots were hybridized with digoxigenin-labelled *DOFT* and *DOFTIP1* specific DNA probes. The size of the DNA markers is given on left in kb.

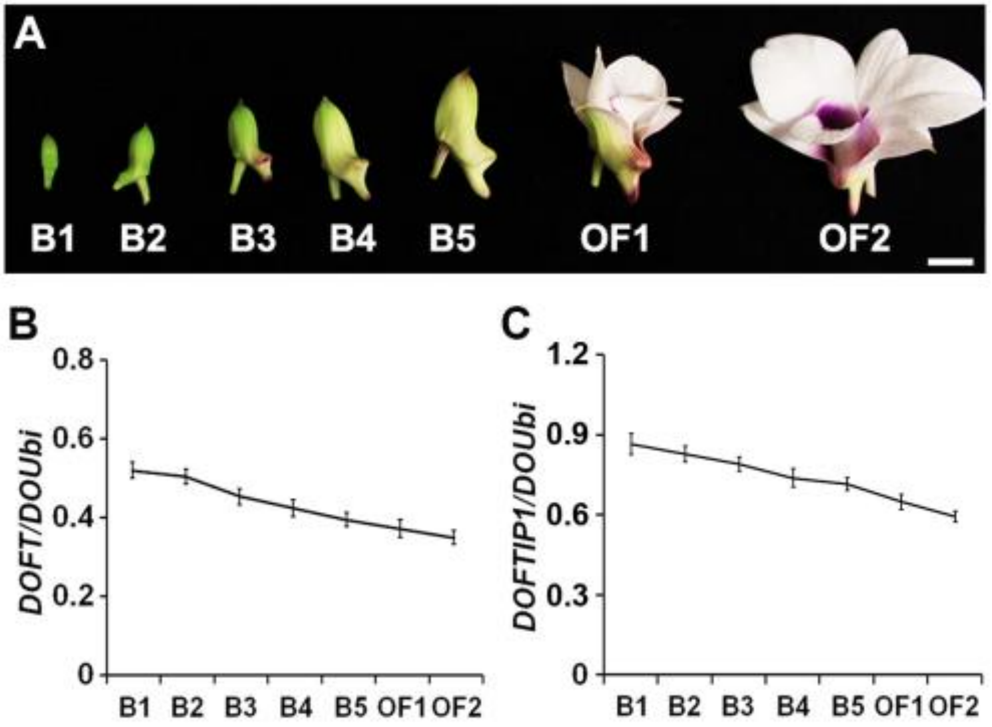

Supplementary Figure S3. Expression of *DOFT* and *DOFTIP1* in flowers at different developmental stages. (A) Different flower developmental stages of *Dendrobium* Chao Praya Smile, including floral buds 1-5 (B1-B5) and open flowers 1 and 2 (OF1 and OF2). Bar = 1 cm. (B, C) Quantitative analysis of the expression of *DOFT* (B) and *DOFTIP1* (C) in flowers at different developmental stages. Error bars indicate SD. Expression levels were determined by quantitative real-time PCR analyses of three independently collected samples. The levels of gene expression were normalized to the expression of the orchid polyubiquitin gene (*DOUbi*).

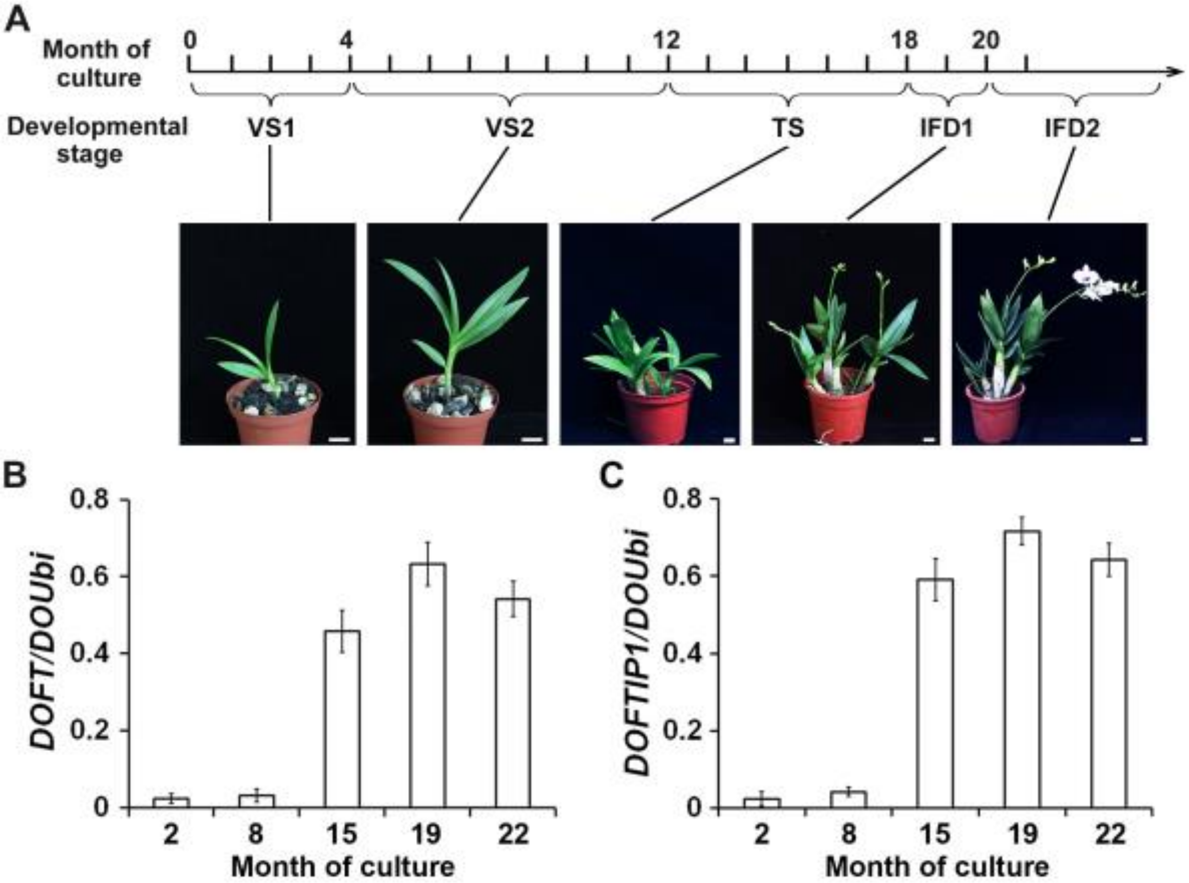

Supplementary Figure S4. Quantitative analysis of temporal expression of *DOFT* and *DOFTIP1* in *Dendrobium* Chao Praya Smile grown under the normal greenhouse condition. (A) Main developmental stages of *Dendrobium* Chao Praya Smile grown under the normal greenhouse condition. The starting materials are young plantlets about 3 cm in height. VS1, vegetative stage 1; VS2, vegetative stage 2; TS, floral transition stage; IFD1, inflorescence and flower development with flower buds; IFD2, inflorescence and flower development with open flowers. Bars = 1 cm. (B, C) Quantitative analysis of the expression of *DOFT* (B) and *DOFTIP1* (C) in *Dendrobium* Chao Praya Smile at different developmental stages under normal greenhouse conditions. Error bars indicate SD. Expression levels were determined by quantitative real-time PCR analyses of three independently collected samples. The levels of gene expression were normalized to the *DOUbi* expression.

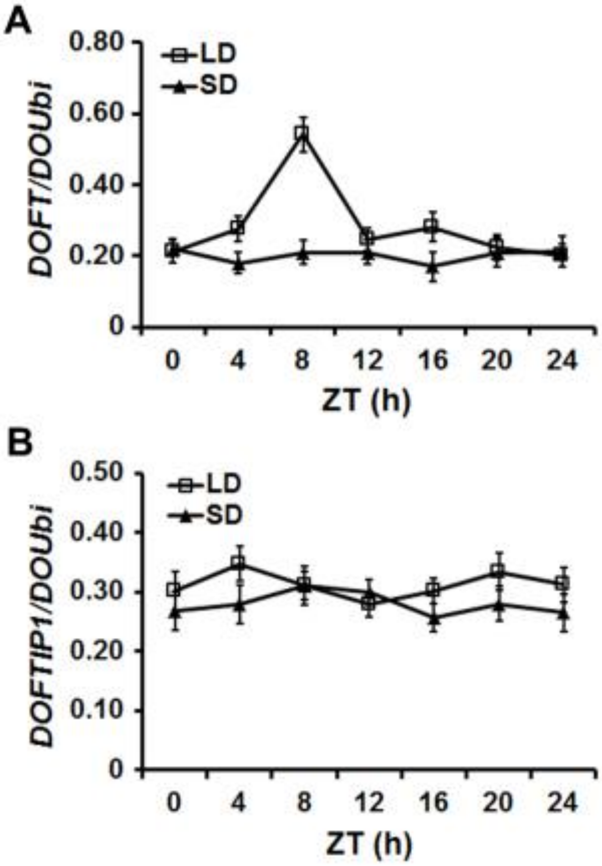

Supplementary Figure S5. Quantitative analysis of *DOFT* (A) and *DOFTIP1* (B) expression levels in leaves of *Dendrobium* Chao Praya Smile within a 24-h cycle under long days (LDs) and short days (SDs). 15-month-old wild-type plants at the transitional stage grown under the normal greenhouse condition were further cultured in a growth chamber under LDs and SDs. After one month, leaves were harvested at 4-h intervals over a 24-h period. Sampling time was expressed in hours as Zeitgeber time (ZT), which is the number of hours after the onset of illumination. Error bars indicate SD. Expression levels were determined by quantitative real-time PCR analyses of three independently collected samples. The levels of gene expression were normalized to the *DOUbi* expression.

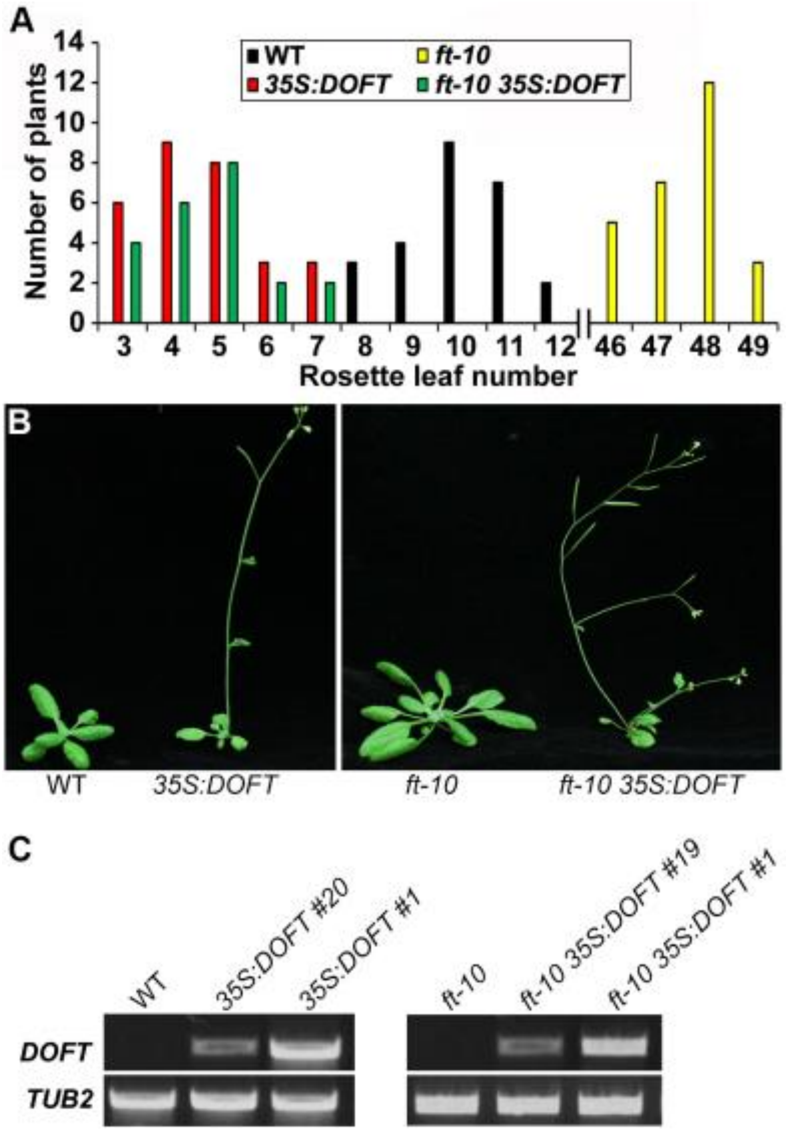

Supplementary Figure S6. Overexpression of *DOFT* promotes flowering in Arabidopsis. (A) Distribution of flowering time in T1 transgenic lines harboring *35S:DOFT* in wild-type and *ft-10* backgrounds under long days. (B) *35S:DOFT* accelerates flowering in both wild-type and *ft-10* backgrounds. *35S:DOFT* shows earlier flowering than a wild-type (WT) plant at 21 days after germination under long days (left panel), while *ft-10 35S:DOFT* shows earlier flowering than an *ft-10* plant at 32 days after germination under long days (right panels). (C) Examination of *DOFT* expression in representative *35S:DOFT* and *ft-10 35S:DOFT* plants by semi-quantitative PCR. *35S:DOFT #1 and ft-10 35S:DOFT #1* exhibit a strong early-flowering phenotype, while *35S:DOFT #20* and *ft-10 35S:DOFT #19* exhibit a weak early-flowering phenotype. The Arabidopsis *TUB2* gene was amplified as a control.

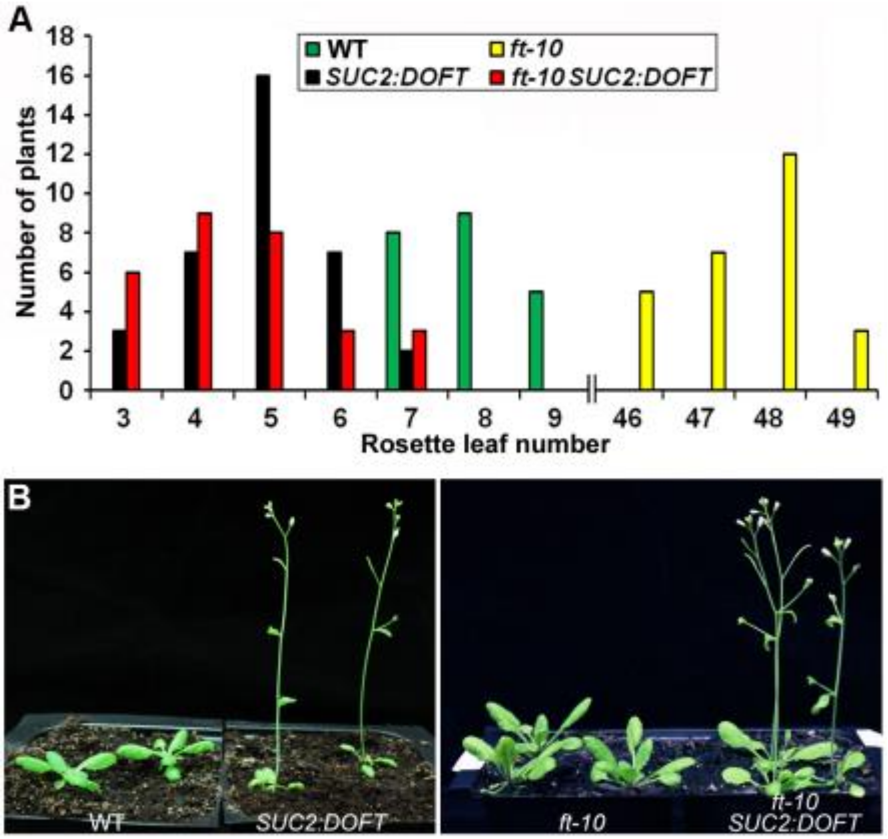

Supplementary Figure S7. Expression of *SUC2:DOFT* promotes flowering in Arabidopsis. (A) Distribution of flowering time in T1 transgenic lines harboring *SUC2:DOFT* in wild-type and *ft-10* backgrounds under long days. (B) *SUC2:DOFT* accelerates flowering in both wild-type and *ft-10* backgrounds. *SUC2:DOFT* shows earlier flowering than wild-type (WT) plants at 21 days after germination under long days (left panel), while *ft-10 SUC2:DOFT* shows earlier flowering than *ft-10* plants at 32 days after germination under long days (right panels).

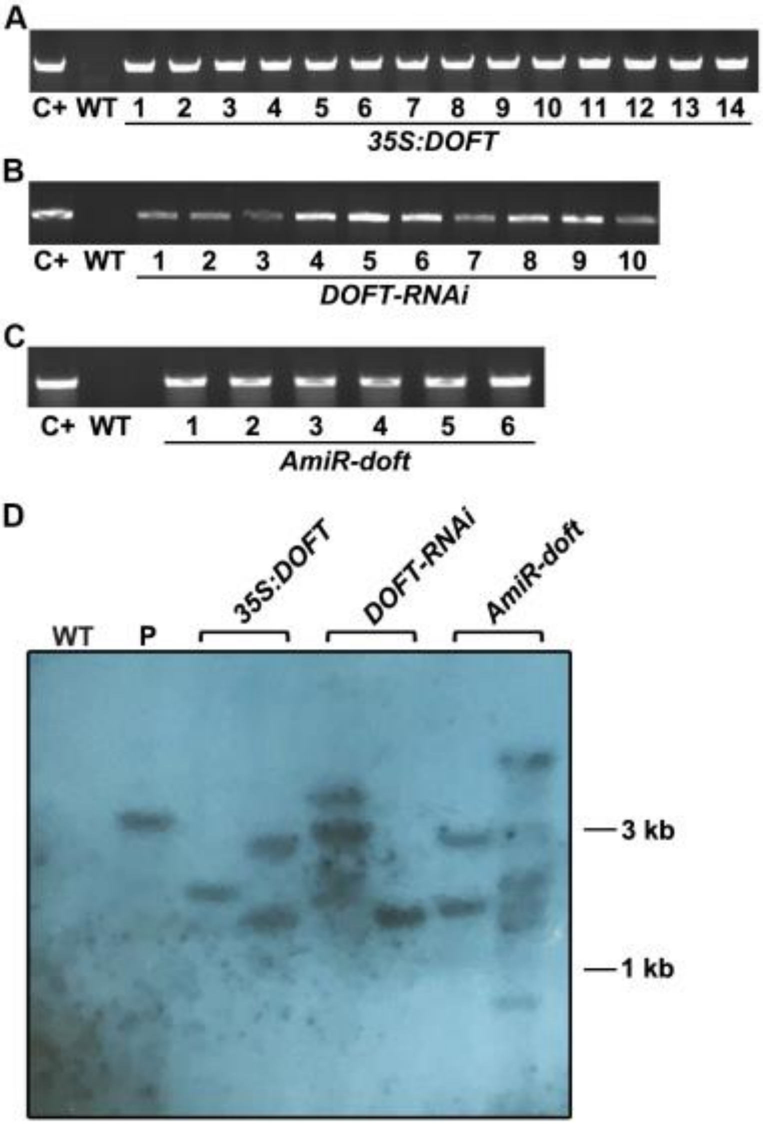

Supplementary Figure S8. Molecular identification of *DOFT* transgenic orchids. (A-C) PCR genotyping of putative *35S:DOFT* (A), *DOFT-RNAi* (B), and *AmiR-doft* (C) transgenic orchids using the primers located within the *35S* promoter and the *DOFT* gene as shown in Supplemental Table 2. The transformation plasmids and genomic DNA from wild-type orchids were served as positive controls (C+) and negative controls (WT), respectively. (D) Southern blot analysis of genomic DNA from representative *DOFT* transgenic orchids. Genomic DNA (20 µg) of transgenic orchids was digested with *EcoR*I and hybridized with the specific probe that detects the *bar* gene in transgenic plants. The genomic DNA from wild-type orchids (WT) and the *pGreen-0229* plasmid containing the *bar* gene (P) were used as negative and positive controls, respectively.

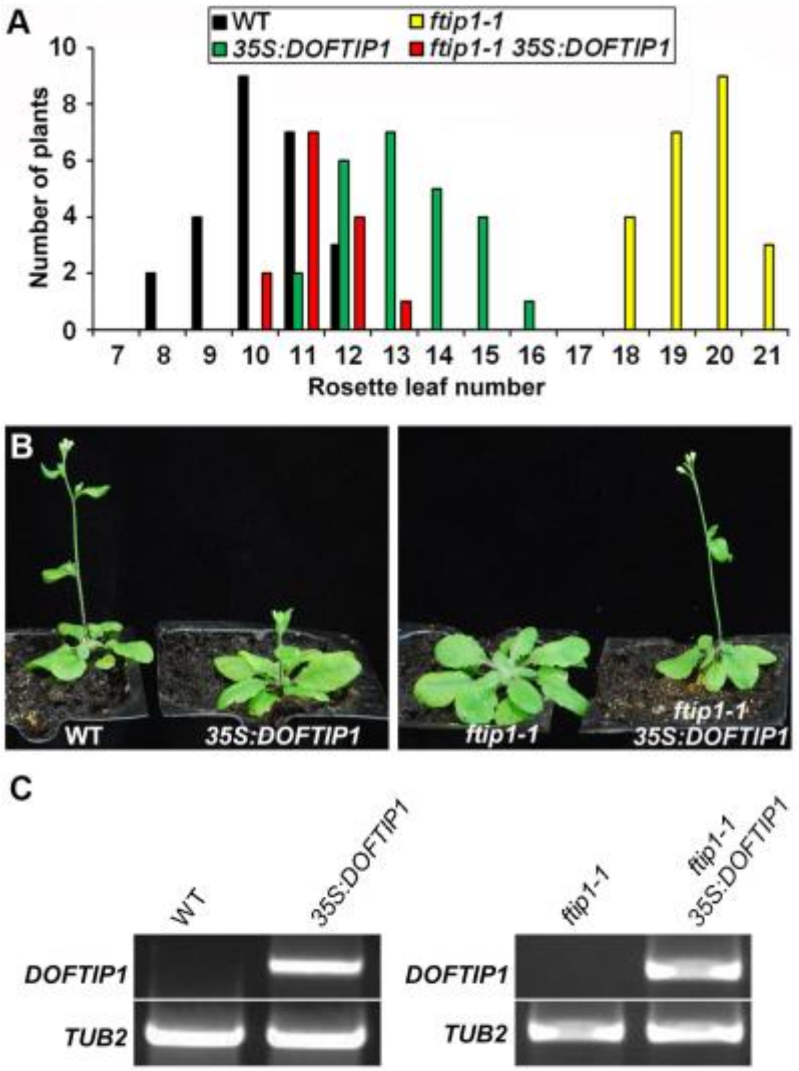

Supplementary Figure S9. Overexpression of *DOFTIP1* rescues the late flowering phenotype of *ftip1-1*. (A) Distribution of flowering time in T1 transgenic lines harboring *35S:DOFTIP1* in wild-type and *ftip1-1* backgrounds under long days. (B) Comparison of flowering time of wild-type, *35S:DOFTIP1*, *ftip1-1*, and *ftip1-1 35S:DOFTIP1* plants at 30 days after germination under long days. (C) Examination of *DOFTIP1* expression in representative *35S:DOFTIP1* and *ftip1-1 35S:DOFTIP1* plants shown in (B) by semi-quantitative PCR. The Arabidopsis *TUB2* gene was amplified as a control.

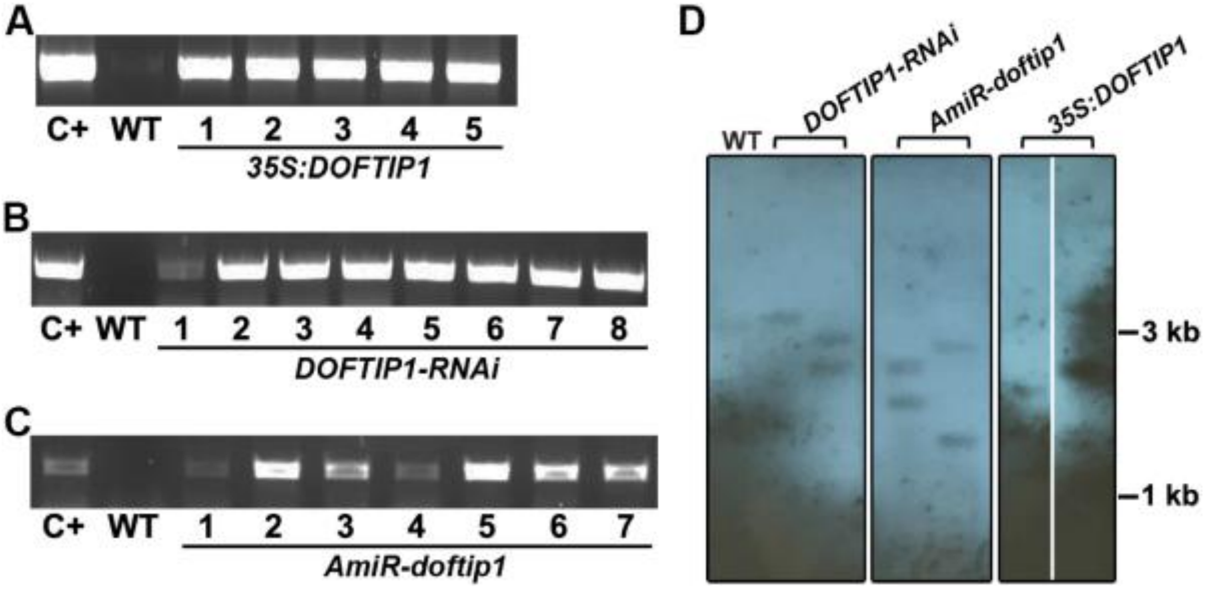

Supplementary Figure S10. Molecular identification of *DOFTIP1* transgenic orchids. (A-C) PCR genotyping of putative *35S:DOFTIP1* (A), *DOFTIP1-RNAi* (B), and *AmiR-doftip1* (C) transgenic orchids using the primers located within the *35S* promoter and the *DOFTIP1* gene as shown in Supplemental Table 2. The transformation plasmids and genomic DNA from wild-type orchids were served as positive controls (C+) and negative controls (WT), respectively. (D) Southern blot analysis of genomic DNA from representative *DOFTIP1* transgenic orchids. Genomic DNA (20 µg) of transgenic orchids was digested with *Eco*RI and hybridized with the specific probe that detects the *bar* gene in transgenic plants. The genomic DNA from wild-type orchids (WT) was used as a negative control.

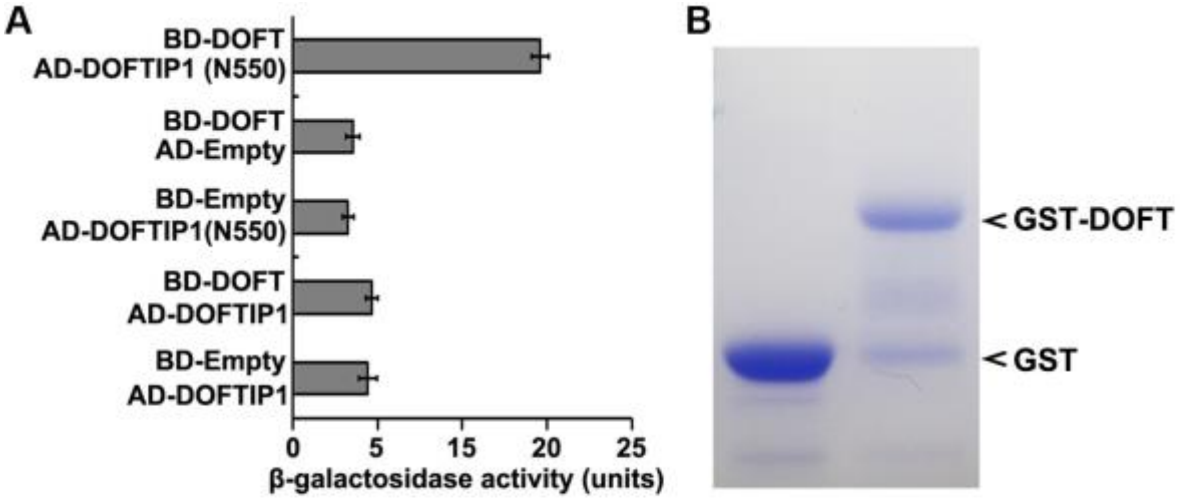

Supplementary Figure S11. DOFTIP1 interacts with DOFT. (A) Quantification of the interaction between DOFT and DOFTIP1 (N550) in yeast cells by β-galactosidase assays. (B) GST-DOFT is expressed in *E.coli* in the presence of 0.6 mM IPTG.

Supplementary Table S1. List of primers used in this study.

Primer pairs used for gene isolation

Gene name	Primer name	Primers (5' to 3')
DOFT middle fragment	DOFT-DF<br>DOFT-DR	AGGAMYTTCTAYACTYTRGT<br>GGRATATCVGTVACYAACCARTG
DOFT 3' RACE	DOFT-3F1<br>DOFT-3F2	TATCCCAGCAACGACCAACGCATCA<br>CGGTCGCCGCCGTCTATTTCAACTG
DOFT 5' RACE	DOFT-5F1<br>DOFT-5F2	CAAATGATGCGTTGGTCGTTGCTGG<br>TGATGCGTTGGTCGTTGCTGGGATA
DOFT full-length	DOFT-F<br>DOFT-R	CATGGGGACTCACGCTCAC<br>CAAAGTACTTGCCAGCAGTGG
DOFTIP1 middle fragment	DOFTIP1-DF<br>DOFTIP1-DR	TTTGTNGCWGCNGARCCWTT<br>TCCCANGTRTAYTGYTCATT
DOFTIP1 3' RACE	DOFTIP1-3F1<br>DOFTIP1-3F2	TCCTCCTCGCTGCTTAACAT<br>GCAATTCATGGTCCATTCAAG
DOFTIP1 5' RACE	DOFTIP1-5F1<br>DOFTIP1-5F2	CCCAACCCTGTCTTCAACACTTATG<br>ACGCTTCATCTGCTTGAGTGCCCAT
DOFTIP1 full length	DOFTIP1-F<br>DOFTIP1-R	ATGATGCAGAGGCCTCTTCGCC<br>TAACATACAATCTGTCCTAGC

Primers pairs used for quantitative real-time PCR

Gene name	Primers (5' to 3')
DOFT	CAAGGAGCGTTTCTCTCAGGG<br>TCACTTGGACTTGGAGCATCTG
DOFTIP1	TGAGGCACCAAGCTACTCAG<br>TGACAGTCCGCATCAAGCAT
DOAP1	CTGTGATGCTGAAGTTGCT<br>CGACCAGTTTGTATTGACG
DOSOC1	CGGCAAGCTCTACGAGTTCT<br>AGCAGGATTCCAGGTTTTCA
DOUbi	GATGGTCGGACTTTAGCGGA<br>GCTGCACAGGTGGAATACCT

TUB2	ATCCGTGAAGAGTACCCAGAT<br>AAGAACCATGCACTCATCAGC

Primers pairs used for probe synthesis for Southern blot analysis

Gene name	Primers (5' to 3')
DOFT	AGAGACCCTTTGATCGTAGGAAGA<br>CCAAAGTATAGAAAGTCCTGAGATC
DOFTIP1	ATGTATTTACTGCCAATGCTGCC<br>TGATTGAACCACTTTCCAACTGC
bar	ATGAGCCCAGAACGACGCCCG<br>TCAGATCTCGGTGACGGGCAG

Primers pairs used for PCR genotyping of putative transgenic orchids

Primer name	Primers (5' to 3')
35S promoter	GACCCTTCCTCTATATAAGGAAGTTC
DOFT_SR	CAAAGTACTTGCCAGCAGTGG
DOFT_RNAi_R	GTCTGCAATAATCCCGCTTAAC
DOFTIP1_R1	CTTCATCTGCTTGAGTGCCCATC
DOFTIP1_RNAi_R	TAGTCTGCAATAATCCCGCTTAAC

Supplementary Table S2. Comparison of flower development in wild-type and transgenic *Dendrobium* Chao Praya Smile after in vitro culture under our growth conditions.

Genotype	No. of Plants	No. of normal inflorescences	No. of abnormal inflorescences/ percentage	Normal flowers	Incomplete or abnormal flowers /percentage
Wild type	26	26	0 (0%)	24	8 (25%)
35S:DOFT	14	11	3 (22%)	6	5 (45%)
DOFT-RNAi	8	3	5 (62.5%)	3	2 (40%)
AmiR-doft	6	3	3 (50%)	2	1 (33.3%)
35S:DOFTIP1	5	5	0 (0)	4	1 (20%)
DOFTIP1-RNAi	8	8	0 (0)	6	2 (25%)
AmiR-doftip1	7	7	0 (0)	6	1 (12.8%)
